# Optimal Allocation of Resources in Female Sex Worker Targeted HIV Prevention Interventions: Model Insights from *Avahan* in South India

**DOI:** 10.1371/journal.pone.0107066

**Published:** 2014-10-01

**Authors:** Jasmina Panovska-Griffiths, Anna Vassall, Holly J. Prudden, Aurélia Lépine, Marie-Claude Boily, Sudha Chandrashekar, Kate M. Mitchell, Tara S. Beattie, Michel Alary, Natasha K. Martin, Peter Vickerman

**Affiliations:** 1 Social and Mathematical Epidemiology Group, Department of Global Health and Development, London School of Hygiene and Tropical Medicine, London, United Kingdom; 2 Department of Mathematics, University College London, London, United Kingdom; 3 School of Social and Community Medicine, University of Bristol, Bristol, United Kingdom; 4 School of Public Health, Imperial College, London, United Kingdom; 5 Centre de recherche du CHU de Québec, Québec, Canada; 6 Département de médecine sociale et préventive, Université Laval, Québec, Canada; 7 St. John’s Research Institute, Bangalore, India; Politehnica University of Bucharest, Romania

## Abstract

**Background:**

The *Avahan* programme has provided HIV prevention activities, including condom promotion, to female sex workers (FSWs) in southern India since 2004. Evidence suggests *Avahan* averted 202,000 HIV infections over 4 years. For replicating this intervention elsewhere, it is essential to understand how the intervention’s impact could have been optimised for different budget levels.

**Methods:**

Behavioural data were used to determine how condom use varied for FSWs with different levels of intervention intensity. Cost data from 64 *Avahan* districts quantified how district-level costs related to intervention scale and intensity. A deterministic model for HIV transmission amongst FSWs and clients projected the impact and cost of intervention strategies for different scale and intensity, and identified the optimal strategies that maximise impact for different budget levels.

**Results:**

As budget levels increase, the optimal intervention strategy is to first increase intervention intensity which achieves little impact, then scale-up coverage to high levels for large increases in impact, and lastly increase intensity further for small additional gains. The cost-effectiveness of these optimal strategies generally improves with increasing resources, while straying from these strategies can triple costs for the same impact. Projections suggest *Avahan* was close to being optimal, and moderate budget reductions (≥20%) would have reduced impact considerably (>40%).

**Discussion:**

Our analysis suggests that tailoring the design of HIV prevention programmes for FSWs can improve impact, and that a certain level of resources are required to achieve demonstrable impact. These insights are critical for optimising the use of limited resources for preventing HIV.

## Introduction

HIV infection remains a global health issue [1]. Many HIV cases occur in settings with low HIV prevalence, such as India [1], where HIV transmission is thought to be driven by high-risk groups (HRGs) [2]–[9]. In 2003, the Bill & Melinda Gates Foundation initiated the *Avahan* India AIDS Initiative, the largest HRG-targeted HIV prevention intervention in the world [10]–[11]. *Avahan*’s aim in targeting female sex workers (FSWs) was to increase their consistency of condom use, and as a consequence reduce HIV transmission between FSWs and clients, and subsequently the general population. The strategy had three main objectives: (a) promotion of safer sex behaviour through peer-mediated communications strategies; (b) increased distribution and social marketing of condoms and management of sexually transmitted infections (STI); and (c) enhancing the enabling environment for the adoption of safer sex practices. *Avahan* programme activities began in January 2004, reaching most districts by mid-2005, with more than 75% of the estimated target population of FSWs contacted monthly by December 2008 [11].

*Avahan* funding included a large-scale programme evaluation. Specifically, a series of district level cross-sectional integrated behavioural and biological surveys (IBBAs) were conducted [12]–[13]. The evaluation used these datasets with HIV transmission models to assess *Avahan*’s impact. Evaluation studies [7], [14]–[17] suggest *Avahan* substantially increased the availability [16], [18] and use [16], [19] of condoms which reduced HIV transmission at population level by 42% averting 202,000 HIV infections over the first 4 years of implementation [16]. Furthermore, a large-scale costing effort established the cost-effectiveness of *Avahan* [20]–[21].

However, *Avahan* required substantial investment ($285 million over 4 years [11]). Given the economic climate [22], and the recent flat-lining of development assistance for health [23]–[24], it remains unclear whether targeted HIV prevention is affordable in India and beyond [25]; and thus the feasibility of sustaining or replicating HIV prevention at scale for HRGs remains uncertain. Emphasis is now being placed on exploring how to reduce the costs of HIV prevention [1], [26] but little is known about how to reduce costs without negatively impacting quality and impact [26]. In addition, the increase in attention being paid to novel prevention technologies, such as anti-retroviral treatment as prevention (TASP) and pre-exposure prophylaxis (PrEP) emphasises the importance of showing that existing effective interventions can be efficiently used to reduce HIV transmission to low levels.

For the first time, this paper illustrates how empirical and model-derived data on costs and impact can inform efficient HIV programme design. Two key characteristics determining the cost and impact of HIV prevention programmes are its scale (numbers of HRG persons reached) and intensity of service delivery (defined by such things as the number of contacts made to each reached person). In this paper we assess how the cost, impact, and cost-effectiveness of *Avahan* were affected by programme scale and intensity. We combine this information to determine whether *Avahan’s* impact could have been achieved at reduced cost if a different intervention scale and intensity had been attained, and whether comparable impact could have achieved with a lower budget.

## Methods

### Overview

A model of HIV transmission between FSWs and their clients (Appendix S1), calibrated to a representative/typical *Avahan* district, was combined with in-depth cost (available from the authors on request) and survey data (freely available from http://ibbainfo.in/) from *Avahan* to explore the relationship between scale and intensity of service delivery, and associated impact (HIV infections averted over 4 years) and costs. To understand the relationship between intensity and impact, we first conducted a regression analysis to quantify how condom use amongst reached FSW with their clients increased with increased intensity (Appendix S2). This relationship was used in the model to estimate the impact achieved at different levels of intervention scale and intensity. In addition, an empirical cost function was used to describe how intervention costs are related to scale and intensity (Appendix S3). The model impact and cost projections were then combined to determine optimal intervention combinations that maximise impact for different budget levels.

### Understanding the relationship between scale, intensity and impact

#### Model description

A dynamic compartmental HIV transmission model amongst FSWs and their clients was developed. The model stratified FSWs by HIV status (infected/uninfected) and whether they were reached by *Avahan* or not. Clients were stratified by HIV status. The modelled HIV intervention programme, based on *Avahan* [10]–[11], increased the consistency of condom use (%CCU defined as the percentage of FSWs that self-reported using of condoms in their last sexual act) between clients and FSWs reached by the intervention (*f*_2_(*t*)).

The model assumed FSWs and clients were infected at a rate proportional to their frequency of commercial sex, consistency of condom use, condom efficacy, probability of HIV transmission per sex act and prevalence of HIV amongst reached and not reached FSWs and clients. FSWs and clients leave the population as they cease commercial sex or die due to AIDS. For simplicity, the population size was kept constant over time. Details on the model equations are provided in Appendix S1. The equations were numerically solved in MATLAB using a Runge-Kutta method.

#### Model parameterisation

The model was parameterised and calibrated to a representative *Avahan* district, Bellary, with 15.6% HIV prevalence amongst FSWs in 2004 (Appendix S1). Uncertainty ranges were defined for the behavioral parameters using data from the IBBA surveys undertaken amongst FSWs and their clients in Bellary [12], [27]–[28], biological parameters came from the published literature [29]–[31], and data on the scale and intensity of intervention activities came from the intervention monitoring system (MIS) [27]. The number of FSWs reached per year in the typical/representative district was set to be the mean annual number of FSWs reached in each district (1429 FSWs) over 2004–2007. Because district-specific mapping size estimates for the number of FSWs were frequently less than this coverage estimate, and in the absence of other suitable size estimates, the estimated number of FSWs in the typical district was proxied by the mean of the maximum number of FSWs reached per year in each district (3200) over 2004–2011. This suggested *Avahan* annually reached on average 45% of FSWs in each district during 2004–2007. The modeled HIV epidemic was assumed to start in 1987 [31].

**Figure 1:**
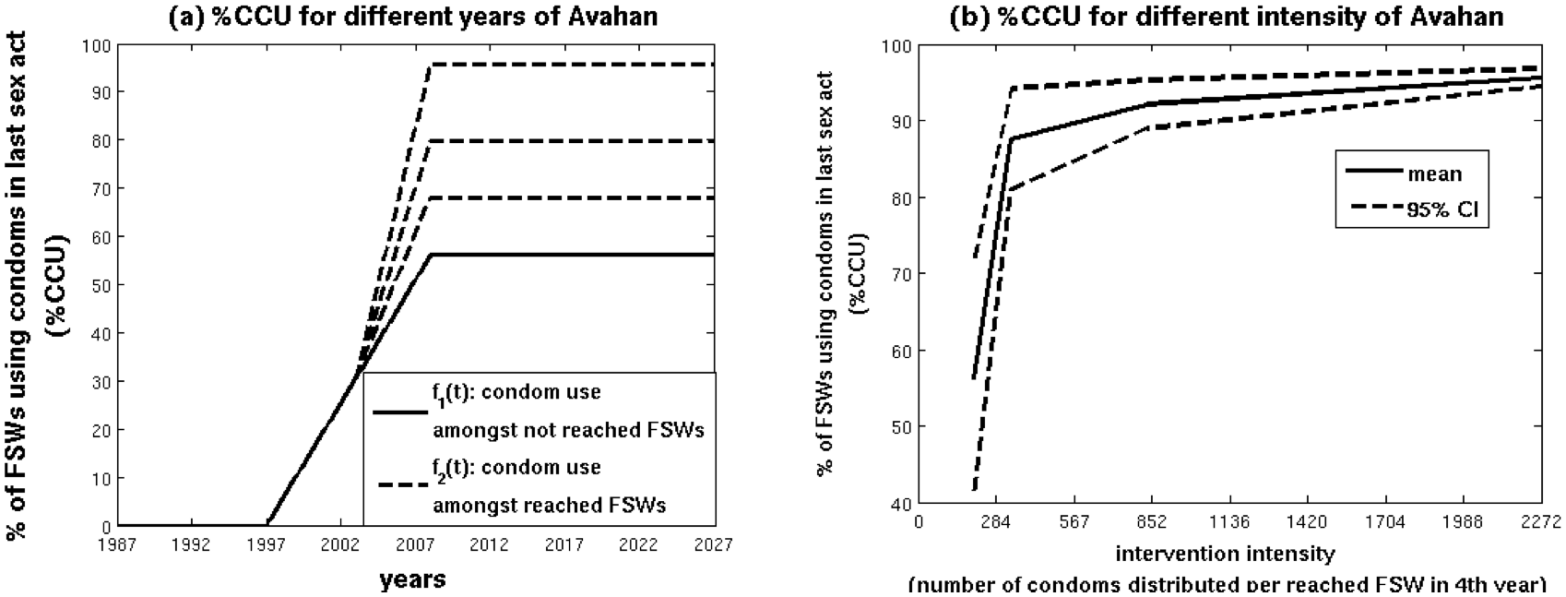
Condom use among FSWs in last sex act before and after the intervention (a), and how condom use amongst reached FSWs in 2008 relates to intervention intensity (average annual number of condoms distributed per reached FSWs in 2008) (b). IBBA data from rounds 1 and 2 amongst FSWs were used to derive these functions. %CCU is the consistency of condom use in the last commercial sex act, CI denotes confidence interval, FSW denotes female sex worker, and IBBA denotes integrated behavioural and biological surveys.

Behavioural parameters, such as the frequency of commercial sex for FSWs and clients, were estimated using round 1 IBBA data for Bellary [12]. In addition, round 1 FSW IBBA data were used to reconstruct a linear time-trend for the increase in condom use among FSWs before *Avahan* (1997–2003, see Figure 1(a)) [12], [19]. Following the start of *Avahan*, condom use was assumed to increase linearly at a greater rate amongst reached FSWs (*f*_2_(t)) than unreached FSWs (*f*_1_(*t*)). The consistency of condom use in 2008 was estimated for reached and unreached FSWs using round 2 (2007/2008) IBBA data [13] from four Karnataka districts, with the consistency of condom use amongst reached FSWs in 2008 being a function of *Avahan’s* intensity or strength of service delivery (Details in Appendix S2). However, because the yearly number of condoms distributed by *Avahan* to each reached FSW (#CD) was the only intervention intensity measure for which a cost function could be estimated (see next section), #CD was the intervention intensity measure used in subsequent model analyses. Importantly, the relationship between %CCU and #CD does not solely represent the effect of distributing more condoms to FSWs, but analysis of the IBBA data confirms it also correlates with other measures of intervention intensity such as the frequency of intervention contacts or educational sessions per reached FSW (Appendix S2). Also, because condoms were available from other sources, %CCU should not be expected to be zero when #CD is zero.

To model the relationship between %CCU amongst reached FSWs in 2008 and #CD, a spline curve was fit to trend data on the level of %CCU for different #CD intervals (Figure 1(b)). Intervention intensity #CD was assumed to range between 0 and 1600 condoms per FSW per year based on the range reported in the MIS for different districts [27]. Lower and higher bounds were also fit to the 95% confidence intervals (CI) of the data shown in Figure 1(b), and were used in the sensitivity analysis (Details in Appendix S2).

The mean intensity reached over all districts for the period 2004–2007 was 267 condoms distributed to each reached FSW per year – this was set to be the intensity achieved in the typical district. However, this was increased 1.4-fold when estimating condom use using the condom use function in Figure 1(b) because the number of condoms distributed per FSW in 2007 from the MIS data was on average 1.4 times greater than the average number distributed over 2004–2007.

#### Model calibration and impact projections

Model calibration involved minimizing [32] the difference between the model simulated and observed HIV prevalence among FSWs (15.6%) and clients (6.2%) from the Bellary round 1 IBBA in 2004. The HIV transmission probability, duration of being a FSW or client and frequency of commercial sex were varied within their uncertainty ranges in order to find a model fit.

For a range of intervention scales (0–3200 FSWs reached over 4 years) and intensities (0–1600 condoms distributed per reached FSW each year), the calibrated model was used to project the impact (HIV infections averted over four years (2004 to 2007) of the *Avahan* intervention due to the increased condom use amongst reached FSW (f_2_(t)) compared to no intervention occurring. The counterfactual ‘no *Avahan*’ scenario assumed condom use amongst FSWs was the same as amongst the unreached FSWs in the IBBA round 2 data (f_1_(t)).

### Understanding the relationship between scale, intensity and cost

Cost data from 64 *Avahan* districts in southern India over 4 years (2004–2007) were used to establish the relationship between the total incremental cost of the *Avahan* intervention (compared to the “no *Avahan*” alternative) and the intensity of service delivery (#CD) and scale of intervention coverage (number of FSWs reached in last year). A panel estimator (a model that uses the complete panel of data collected over each of the four years) was used with fixed effects to derive an empirical equation for the average incremental intervention cost per year over 4 years (Details in Appendix S3).

### Combining cost and impact projections to determine optimal intervention combinations

For different intervention scales and intensities, the model impact projections were combined with the corresponding incremental cost projections to explore the cost, impact and cost-effectiveness (incremental cost per infection averted) of different intervention strategies over 2004–2007 (4 years) compared to if *Avahan* had not occurred. We thus identified different intervention combinations that achieve the same impact for different costs or different impacts for the same cost, thereby allowing us to identify ‘optimal’ intervention combinations that gave the greatest impact for a specific incremental cost.

To assess how efficient *Avahan* was, the estimated incremental cost and impact of achieving *Avahan*’s mean scale and intensity in the representative district was compared to the cheapest intervention combination that achieved the same impact. Alternative scenarios then considered the efficiency associated with the scale and intensity achieved in each *Avahan* district. To assess what impact *Avahan* could have achieved with a reduced budget, the impact associated with the scale and intensity achieved in each district was compared to the optimal impact achievable with 50, 60, 70, 80, 90 and 100% of the budget. These scenario analyses for each district assumed the same ongoing HIV epidemic as the representative district but scaled the FSW population to its estimated size in each district considered. The efficiency analyses undertaken for the representative district were then repeated for the scale and intensity achieved in each district, with the results across districts being combined to produce a median and interquartile range.

### Sensitivity analysis

A sensitivity analysis was undertaken to determine the robustness of the model’s projected relationship for the optimal impact achieved at different budgets. We firstly explored the effect of uncertainty in the relationship between %CCU and intervention intensity or the relationship between incremental cost and intervention scale or intensity. Then we considered the effect of not replacing HIV deaths but having population growth, or of incorporating an initial acute stage of HIV with increased HIV infectivity, or assuming that the *Avahan* intervention started in 2014 (instead of 2003) while incorporating current levels of ART coverage (details in Appendix S1).

## Results

### Relationship between scale, intensity and impact or cost

**Figure 2:**
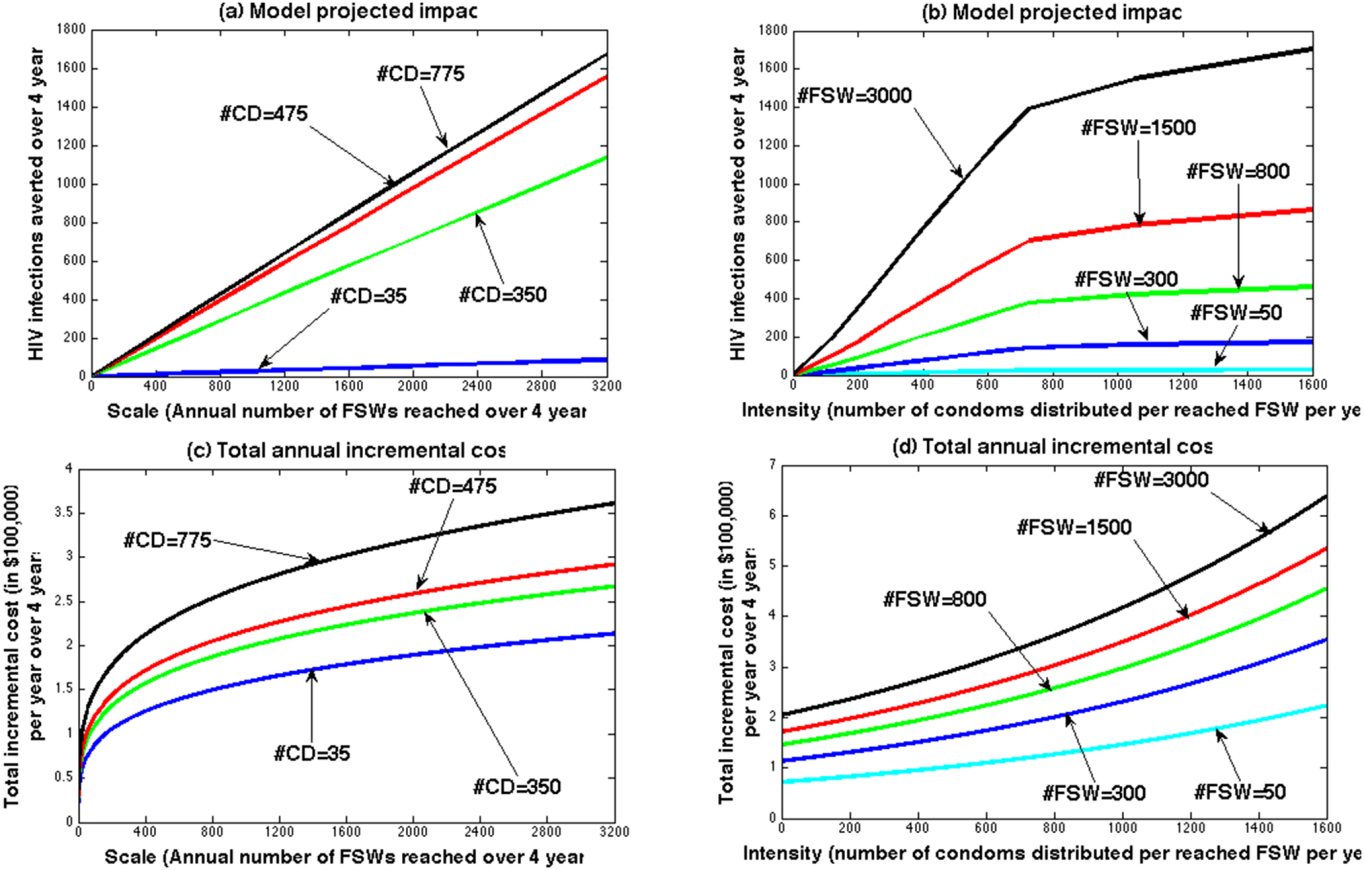
The relationship between total intervention impact and annual cost (in $100,000 per year) over 4 years (2004–2007) and intervention scale (Annual number of FSWs reached by the intervention over 4 years - #FSW) (a and c) or intervention intensity (annual number of condoms distributed per reached FSW over 4 years - #CD) (b and d). Impact is projected by the model (described in main text and Appendix S1) and total cost is estimated by the cost function *TC = scale^a^e* ^10.18+b^*^intensity^ (a = 0.256, b = 0.00071 as per equation (1) of Appendix S3).

The calibrated model (Figure S1 in Appendix S1) projected a consistent and approximately linear increase in impact with increasing scale (Figure 2(a)). The increase in impact with intensity exhibited similar behaviour at low intensity but levelled off at higher intensity (Figure 2(b)). In contrast, the empirical cost analysis (Figure 2(c,d)) found dramatic economies of scale – the marginal cost of reaching an additional FSW decreased as scale increased. For example, it costs $268,000 per year to reach the first 1000 FSWs (#CD = 775), whereas it only cost an extra $52,000 to reach the next 1000 FSWs. There was no evidence for diseconomies of scale at high scale. The marginal cost of increasing intervention intensity was also non-linear and showed initial fixed costs, then roughly linear increases in costs with increasing intensity with slight diseconomies of intensity at high intensity (#CD>600).

**Figure 3:**
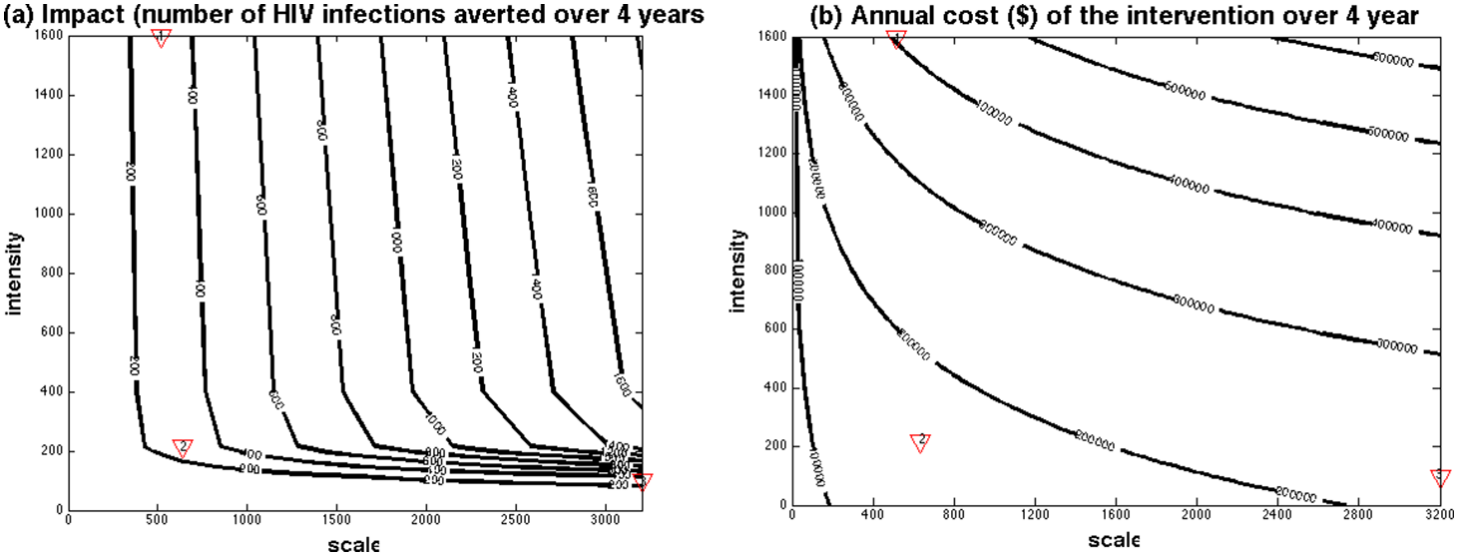
Model-projected intervention impact (a) or total annual cost over 4 years (2004–2007) (b) for different scale (average number of FSWs reached by the intervention each year over 4 years) and intensity (average annual number of condoms distributed per reached FSW over 4 years or #CD). For each contour, the impact (number of HIV infections averted over 4 years) (a) or cost (b) remain the same for different combinations of scale and intensity. The red triangles in the figures refer to intervention examples discussed in the text that all avert 300 HIV infections over 4 years.

**Figure 4:**
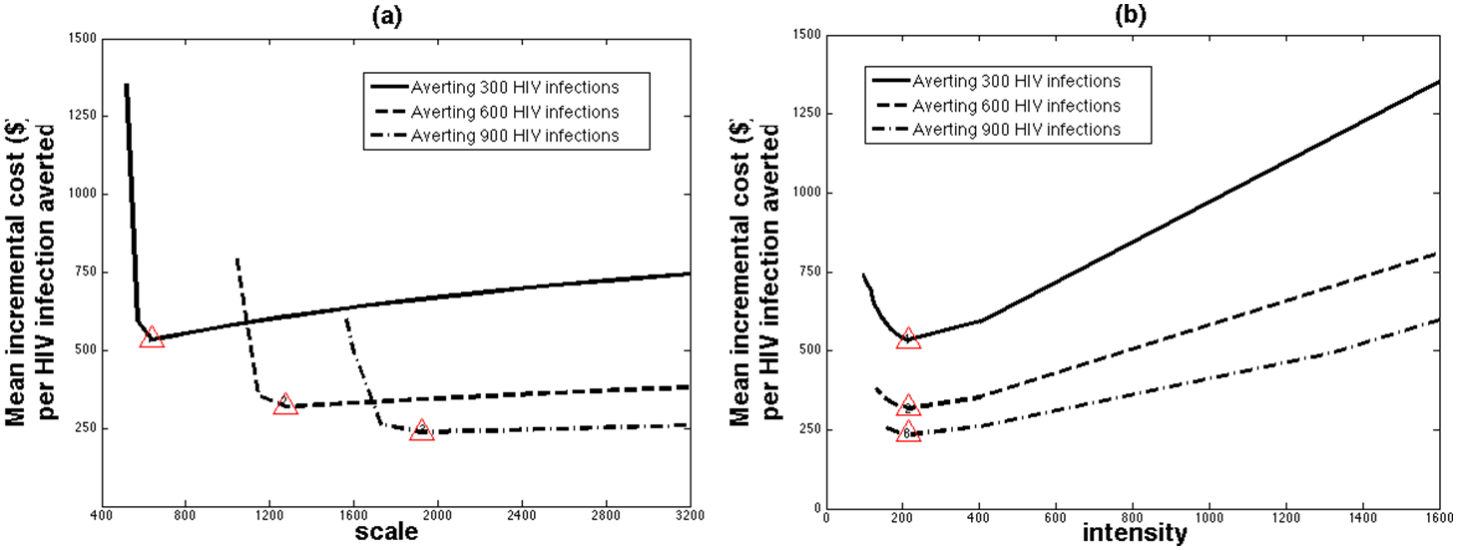
The cost-effectiveness of different modelled intervention combinations that avert 300, 600 or 900 HIV infections between 2004 and 2007 for different levels of scale (average annual number of FSWs reached by the intervention over 4 years) and intensity (average annual number of condoms distributed per reached FSW over 4 years). The optimal intervention for averting 300, 600 and 900 HIV infections is the minima of each curve (the red numbered triangles on each curve; with respective cost-effectiveness of $2136, $1279 and $947 per HIV infection averted) when respective scale is 636, 1278 and 1926 FSWs reached per year and average intensity is #CD = 216. The red triangles in the figures refer to these optimal (scale, cost-effectiveness) (in (a)) and (intensity, cost-effectiveness) (in (b)) combinations.

Figure 3 shows the contours along which the intervention’s impact or incremental cost for the representative district remains constant for different combinations of intervention scale and intensity. Many combinations of intensity and scale can give similar impact (Figure 3(a)) or incremental cost (Figure 3(b)), such that the same impact can be achieved at different incremental costs. For example, 300 HIV infections can be averted over 4 years with a range of intervention scales and intensities (Figure 3(a)- red numbered triangles), with the cost-effectiveness ratio (Figure 4) ranging from $5420 per HIV infection averted when 517 FSWs are reached (#CD = 1600; red triangle 1) down to $2136 for the optimal intervention combination (scale = 636, #CD = 216; red triangle 2) and up to $2976 per HIV infection averted when 3200 FSWs are reached (#CD = 99; red triangle 3). The same projections for 600 and 900 HIV infections averted show similar trends but with it being more cost-effective because of the higher scales needed (Figure 4). These projections also suggest that the cost of achieving 300, 600 or 900 HIV infections averted can differ 3-fold depending on whether the optimal intervention scale and intensity is achieved or not.

### The impact and cost-effectiveness of different optimal intervention strategies

**Figure 5:**
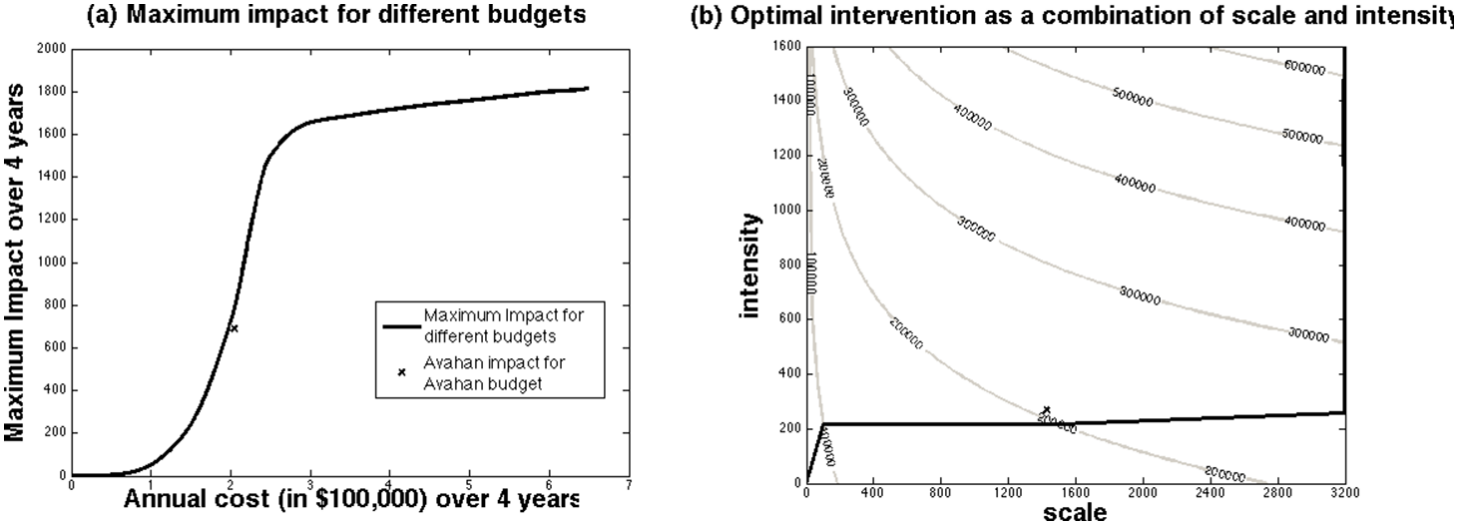
The relationship between incremental annual cost and impact over 4 years (a) or scale and intensity (b) for the optimal intervention strategies that maximise impact for different budget levels. In (a) we projected the maximum intervention impact, as the number of HIV infections averted, for different budget levels between 2004 and 2007. In (b) we show the optimal combination of scale (average number of FSWs reached each year between 2004 and 2007) and intensity (average number of condoms distributed per FSWs per year: #CD) to attain this maximum intervention impact, with contours of constant annual cost over 4 years also shown for reference. The cross in (a) signifies the estimated cost and impact of *Avahan* in the representative district, whereas in (b) it signifies the average scale and intensity of *Avahan* in the representative district.

The maximum impact achievable at different budget levels is illustrated in Figure 5(a) with the associated *optimal* combination of scale and intensity being shown in Figure 5(b), with contours of equal annual incremental cost also shown for illustration. Figure 5(a) shows that below an annual budget of $100,000 or $31 per FSW, little impact can be achieved over 4 years, with the optimal intervention having low scale but increasing intensity (Figure 5(b)). With further increases in annual budget to $300,000 or $94 per FSW, the maximum impact at each budget level increases rapidly to over 1600 HIV infections averted over 4 years at $300,000 per year. To achieve this increase in impact, the optimal intervention strategy requires large increases in scale with intensity remaining stable (#CD = 216). Once the annual budget exceeds $300,000 or over $94 per FSW, scale is largely maximised and further increases in budget are used to further increase intensity with little additional impact.

**Figure 6:**
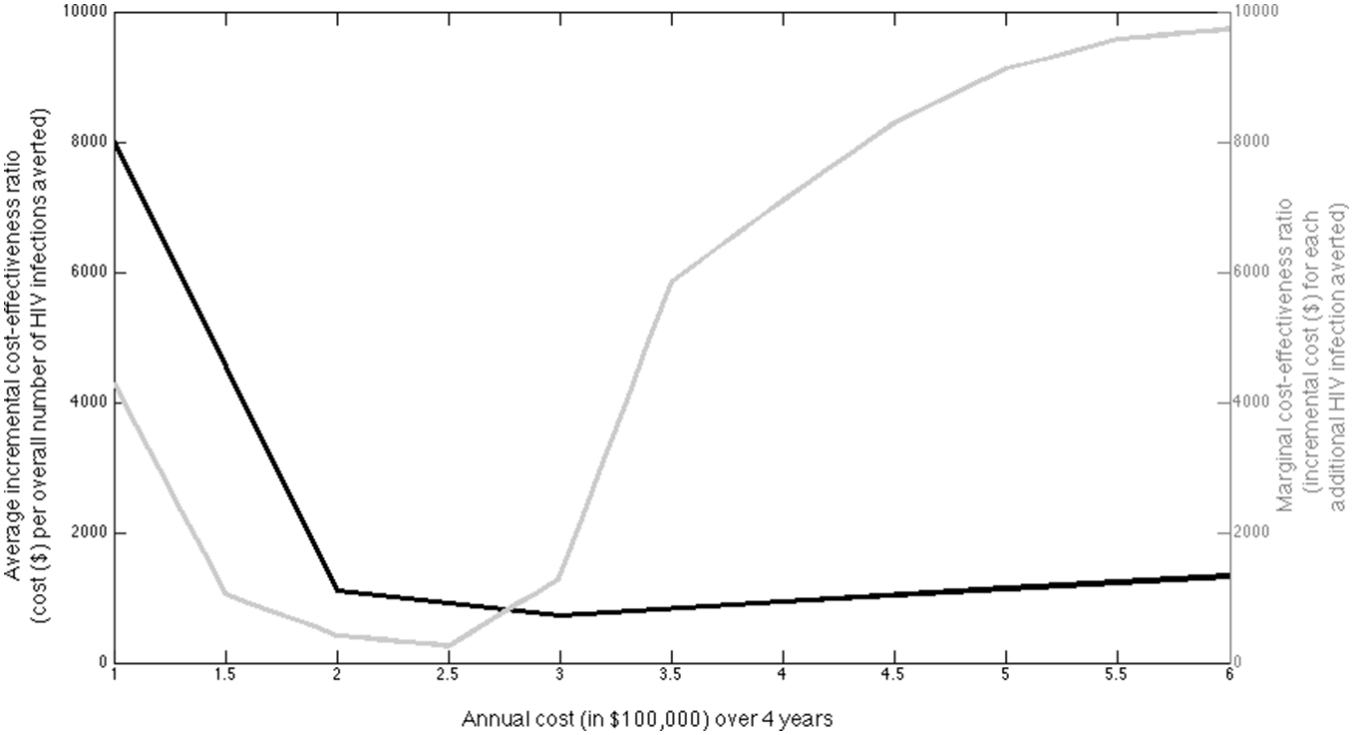
Illustration of the relationship between yearly budget levels (averaged over 4 years of intervention period (2004–2007)) and both the average incremental cost-effectiveness ratio (*ICER = cost*/*impact*; dark-grey curve) and the marginal cost-effectiveness ratio (MCER = Δ*cost*/Δ*impact*; light-grey curve) for each additional HIV infection averted when the optimal intervention combination is adhered to in the typical/representative district.

Figure 6 shows the relationship between budget level and both the mean incremental cost-effectiveness ratio (ICER) compared to the ‘no-Avahan’ alternative for each budget level, and the marginal change in the ICER per HIV infection averted as budgets increase, both for when the optimal intervention combination is adhered to. Commonly, economic evaluations are conducted for a particular intervention with specified scale; a constant ICER is estimated that is then compared to a willingness to pay threshold to determine if the intervention is cost-effective and should be expanded. Figure 6 suggests that making such a recommendation based on a single ICER for any specific observed/modelled scale of service may be incorrect. Indeed, if the marginal cost-effectiveness ratio (MCER) for each extra HIV infection averted, compared to the no-*Avahan* alternative increases beyond a specified willingness to pay threshold, then the intervention should not exceed that scale if it is to remain efficient, and adoption at beyond this scale should not be recommended. As a direct result of the non-linear relationship between incremental cost and impact in Figure 5(a), the mean ICER for the optimal intervention combination is initially very high for annual budget levels below $100,000 ($8,000 per HIV infection averted if spend $100,000 annually), but then decreases rapidly for annual budgets between $100,000 and $300,000 ($720 per HIV infection averted if spend $300,000 annually) and then increases for budgets over $300,000 ($1320 per HIV infection averted if spend $600,000 or $187 per FSW annually). As the ICER rises, the MCER also begins to rise rapidly. However, the MCER is still likely to remain below the Indian willingness to pay threshold (around US$1500 per DALY if ∼7.5 DALYs averted per HIV infection averted as estimated in the cost-effectiveness analysis of the Avahan intervention) for the budget range considered in Figure 6.

### Could the impact of *Avahan* have been achieved at lower cost?

If we apply the relationships in Figure 3 to the average scale (1429 FSWs reached annually) and intensity (#CD = 267) reached across *Avahan* districts over 2004–2007, this translates to a projected 691 HIV infections averted in the representative district at an annual incremental cost of $204,710 ($64 per FSW). For this budget level, the model suggests the same impact could have been achieved with $22,720 (3%) less budget over 4 years (scale of 1472, #CD = 216). This increases to a median 6% (IQR 3–14%) reduction in budget when we analyse each district separately. This reduction in incremental cost is generally achieved through optimising intensity in each district (to #CD = 216) and adjusting scale accordingly to maintain impact, with 28/63 districts increasing intensity while decreasing scale and the remainder doing the opposite.

Conversely, if the programme had been implemented with optimal intensity and scale but with less budget, then our projections suggest *Avahan* would have achieved markedly less impact for budgets below 90% of what is currently available ($58 per FSW), with a median 9, 17, 32, 53, 84 or 126% of the current estimated impact being achieved if 50, 60, 70, 80, 90 or 100% of the current budget was available, respectively. In most districts, these reductions in budget resulted in reduced scale, with stable intensity, which as shown in Figure 5 are associated with massive reductions in impact and diminished cost-effectiveness (Figure 6).

### Sensitivity analysis

All our sensitivity analyses (Figures S2–S4 in Appendix S1) suggest the same relationship between intervention scale and intensity for the optimal interventions that maximise impact at different budgets (Figure 5(b)), with the optimal strategy still being to first increase intensity, then maximise scale and lastly increase intensity again. However, although the same qualitative relationship always occurs between impact and budget, some scenarios achieve substantially less (or more) impact for the same cost (and scale/intensity combination), such as the scenarios including the acute phase of HIV with or without the inclusion of ART. These two scenarios achieve less impact because the inclusion of the HIV acute phase results in a more rapid HIV epidemic for the same ultimate HIV prevalence, and so when the intervention starts the epidemic is already in decline (due to pre-intervention increases in condom use) with lower HIV incidence causing less HIV infections to be averted by the same intervention. Importantly, despite these differences all the sensitivity analyses suggest a very similar small reduction in budget (1.9–3.6%) could have been possible in the *Avahan* typical/representative district without reducing impact.

## Discussion

In this paper, empirical cost and survey data collected for the *Avahan* evaluation are combined with mathematical modelling to explore how intervention scale and intensity of service delivery should be allocated to maximise impact for the resources available. It is the first time that empirical cost functions have been explicitly combined with impact modelling projections to assess the relationship between cost and impact of different targeted HIV prevention programme designs: not simply asking was the intervention cost-effective, but also whether the most efficient strategy was adopted.

Our analysis produces insights of relevance to evaluating the efficiency of *Avahan*, and for replicating similar interventions elsewhere. The results suggest that as budget resources increase, the focus of a FSW-targeted condom promotion intervention should change, with efforts initially focussing on achieving a minimum level of intensity, to then scaling up this intervention package to high coverage, and then increasing intensity again. The impact achieved is also very sensitive to the resources available, with a minimum level of resources being needed to achieve a non-negligible impact (>3% of all HIV infections averted) – this is $31 per estimated FSW in the setting per year for a population of 3200 FSWs and $57 or $12 per FSW per year for a population of 1000 or 10,000 FSWs, respectively. These findings are driven by the non-linear relationships between costs, impact, scale and intensity, especially the considerable economies of scale, the contrasting diseconomies with increasing intensity, and the plateauing relationship between intervention intensity and condom use. This means that investing resources in improving intensity achieves little beyond a certain threshold as compared to increasing scale. The cost-effectiveness ratio of the intervention also changes as these shifts in priority occur, with large increases in impact and improvements in the cost-effectiveness ratio occurring with initial increases in scale and budget, but with this tailing off as scale is maximised. Additionally, our analysis suggests that if *Avahan* had adopted this optimal approach, then at best it could have saved 6% of its costs while still maintaining the same impact, suggesting that *Avahan* was close to being optimal in its intervention design. However, in contrast 26% greater impact could have been achieved with the same budget if Avahan had reached higher scale across all districts. Unfortunately, reducing the budget much below current levels could dramatically reduce impact, with half the impact being achieved with 80% of the budget ($51 per FSW) and little impact being achieved with half the budget ($32 per FSW). This again highlights the importance of defining minimum budget levels for achieving any impact.

Our results suggest that for most budget levels, the optimal intervention intensity is proxied by annually distributing approximately 216 condoms per reached FSW. This is not the number of condoms used by each FSW because condoms are available from elsewhere, but correlations with other intensity measures (within the IBBA data) suggest it translates roughly to a reached FSW being contacted once a week by a peer-worker and receiving prevention educational sessions twice per month. Interestingly, this compares well with the Indian National AIDS Control Organisation (NACO) targets of reaching each FSW twice per month [33]. In terms of cost, this intervention intensity translates to annually spending $213 per reached FSW for a program reaching 800 FSW, or $127 if 1600 FSW are reached. These cost estimates are 5-fold more expensive than NACO budget guidelines [33] of $29–43 per year per reached FSW for FSW interventions reaching similar scale. However, we note that these guideline costs do not include the costs that occur above the NGO level [34] which make up 65% of Avahan’s total costs, so making our cost projections comparable to the NACO guidelines [33] if we remove the above NGO costs from our estimates (65% of our estimated $127 per reached FSW).

This work adds to the current literature base highlighting the importance of exploring non-linear cost functions for guiding HIV prevention resource allocation and programme design [34]–[37]. The analysis is unique in that it also determines how impact is affected by the way resources are allocated, and so derives a combined non-linear ‘cost-effectiveness’ function. Applying such an approach broadly has been, and will remain a challenge [34], [37], primarily because existing resource allocation and efficiency studies for HIV interventions have not had the data to determine these key relationships. Previous models and studies therefore have tended to consider impact and cost in isolation [25]; and commonly, the average costs per person reached are defined as constant over scale and scope [25], [37]. This study suggests that non-linear functional forms [36], [37] for both costs and impact should be considered in resource allocation decision making if optimal allocation is to be achieved. Moreover, we have demonstrated that empirically exploring the relationships between cost, impact and important programme design characteristics has the potential to improve programme implementation. While scale and intensity are key considerations, exploring how costs and impact relate to investments in community mobilisation and other extensions of programme scope may also be considered. Our work represents the first step in a study on how best to allocate resources for large-scale combination HIV prevention interventions among HRGs. Our findings are of use for understanding what level of intervention intensity is required when scaling up intervention activities to other districts and states of India and possibly elsewhere. However, it is important to note that because this analysis was the first of its kind, other similar analyses need to be undertaken that improve and add to what was done here especially with respect to considering other measures or aspects of intervention intensity. The next steps will be to extend the analysis to consider multiple HRGs, interventions and epidemic settings, and how resources should be allocated between them depending on the budget available.

One of the limitations of our approach is the simplicity of the model used. This was done because greater complexity was deemed unnecessary for achieving the study aims. Sensitivity analyses suggest the model’s simplicity did not adversely affect the model projections. Incorporating other behavioural heterogeneities is also unlikely to have affected the model findings [38]. Future analyses considering multiple interventions, HRGs and settings will use more complex models. Moreover, the data used for our analyses had some weaknesses including the reliance on programmatic data. In particular, the choice of intensity measure was determined by the limited outcome measures recorded in the intervention monitoring system (MIS). The MIS did not include reliable data on other service intensity measures such as the number of contacts or education sessions per FSW, and so the number of condoms distributed per FSW was used as a proxy intensity measure. Analysis of the IBBA data confirms the validity of this assumption - FSWs that obtain more condoms are also likely to have more peer-educator contacts and educational sessions. Limitations also exist in the use of self-reported behavioural data, particularly the level of condom use for different levels of intervention intensity. Although self-reported levels of condom use are likely to over-estimate real levels of use, it is less likely that they will affect the observed qualitative relationship between condom use and intervention intensity. Also, other modelling undertaken by our group suggest the observed increases in self-reported condom use are consistent with observed changes in HIV prevalence in most districts lending support to our use of this data [7], [14], [16]. Also, district-specific FSW size estimates were frequently below the scale achieved by *Avahan* in that setting, and therefore the size of the FSW population in each district was estimated by the maximum scale of *Avahan* over the period 2003–2011. Although this could underestimate the real FSW population size for each district, it should not change our general projections because few districts were approaching full scale.

Another limitation of our analysis is that we focussed on a representative district rather than considering all Avahan districts, with the issue of generalisability being approached through undertaking sensitivity analyses. The drawback of this approach is that we did not explicitly model the HIV epidemic in all 63 districts, something that was not possible because limited data was available from many districts. Although the actual impact will vary by district, additional sensitivity analyses have shown that the qualitative relationship between coverage and intensity for maximising impact remains unchanged, and hence our approach is sufficient for exploring whether the allocation of resources in districts with different size and HIV prevalence has been undertaken optimally.

In conclusion, our analysis suggests that tailoring the design of HIV prevention programmes for FSWs can both improve their impact and cost-effectiveness. Designing optimal interventions through careful selection of the most effective combination of services to target FSW is in line with specific targets included in the Joint United Nations Programme on HIV/AIDS (UNAIDS) 2011–15 strategy [39], and is critical to optimising the use of resources for preventing HIV in many countries.

## Supporting Information

Appendix S1 **Description of model equations, parameterisation and sensitivity analysis.**
(DOCX)

Appendix S2 **Description and analysis of the Integrated Biological and Behavioural Assessment (IBBA) survey data to estimate the level condom use among reached FSWs by** ***Avahan*** **for different levels of intervention intensity.**
(DOCX)

Appendix S3 **Relationship between intervention costs, and intervention scale and intensity of service delivery.**
(DOCX)
