# Supplementary material for: Optimal Allocation of Resources in Female Sex Worker Targeted HIV Prevention Interventions: Model Insights from Avahan in South India
Source: PLoS One. 2014 Oct 1;9(10):e107066. doi: 10.1371/journal.pone.0107066 (PMC4182672; doi:10.1371/journal.pone.0107066)
Supplement: Appendix S1 — Description of model equations, parameterisation and sensitivity analysis. (DOCX) [file pone.0107066.s001.docx]

**Appendix S1: Description of model equations, parameterisation and sensitivity analysis**

**A1: Simple baseline model of HIV transmission amongst female sex workers (FSWs) and their clients**

**Model description**

We develop a dynamic compartmental model to describe the transmission of HIV between different populations of female sex workers (FSWs) and their commercial partners (clients). The model distinguishes between FSWs that were reached by the *Avahan*-style HIV prevention intervention [1]-[2] (population ${TOT}_{F2}$ divided into susceptible and infected populations $y_{2},y_{4}$) and those FSWs that were not reached (population ${TOT}_{F1}$ divided into susceptible and infected populations $y_{1},y_{3}$). Clients were only stratified by whether they were HIV infected ($y_{6}$) or not ($y_{5}$), with total population ${TOT}_{C}$. The modelled HIV intervention programme was based on the *Avahan* AIDS Initiative [1-2] and was assumed to increase the consistency or average probability of condom use amongst FSWs with each client. This is defined by time dependent function $f_{2}(t)$ for clients with FSWs reached by the intervention, as compared to the consistency of condom use amongst clients with FSWs not reached by the intervention $f_{1}(t)$.

As in previous models of HIV transmission between FSWs and clients [3-5], the model assumes FSWs and clients are infected at a rate related to their frequency of commercial sex (with the number of commercial sexual partners defined as $c_{1}$ for FSWs and $c_{2}$ for clients), consistency of condom use ($f_{1}(t)$ or $f_{2}(t)$ for unreached and reached FSWs), condom efficacy (e), the probability of HIV transmission per sex act from male to female ($\beta_{MF}$) or from female to male$( \beta_{FM}$) and the prevalence of HIV among reached and not reached FSWs ( $p_{FSW}$) and clients ${(p}_{C})$. FSWs and clients leave the population as they cease commercial sex at respective per capita rates $\alpha_{1}$ and $\alpha_{2}$, and infected FSWs and clients also leave the population due to death from AIDS at a rate $\eta$. For simplicity, the FSW and client populations were assumed to remain constant over time, with all individuals ceasing commercial sex or dying from AIDS being replaced with susceptible individuals of the same risk group (reached/unreached FSWs or clients). Although this assumption may seem unrealistic, it is unlikely to affect the projections much because sex work is quite short lived in these settings and the HIV prevalence is not too high [1-2]. Our sensitivity analysis explores the implications of this assumption. To balance the number of sex acts had by FSWs and clients we imposed the following condition $c_{1}{TOT}_{F1}+c_{1}{TOT}_{F2}=c_{2}{TOT}_{c}$ which was then used to estimate the number of clients (Table S1).

The differentiating factor between FSWs reached or not by the intervention, are the functions $f_{2}(t)$ and $f_{1}(t)$, which describe the consistency of condom use among FSWs reached by Avahan or not. These functions are the same before *Avahan*, but then diverge after *Avahan,* with $f_{2}(t)$ then being generally greater than $f_{1}(t)$ with its magnitude being related to Avahan’s intensity of service delivery (Figures 1(a)-(b) of the main text). This is described more in the main text of the paper and in Appendix S2.

The system of equations that comprise our model is:

$\frac{dy_{1}}{dt}={\alpha_{1}}{TOT}_{F1}+\eta y_{3}-\beta_{MF}c_{1}\left( 1-ef_{1} \right)y_{1}\frac{y_{6}}{{TOT}_{c}}-{\alpha_{1}}y_{1}$ Susceptible not reached FSW (1)

$\frac{dy_{2}}{dt}={{{\alpha_{1}}}TOT}_{F2}+{\eta y}_{4}-\beta_{MF}c_{1}\left( 1-ef_{2} \right)y_{2}\frac{y_{6}}{{TOT}_{c}}-{{{\alpha_{1}}}y}_{2}$ Susceptible reached FSW (2)

$\frac{dy_{3}}{dt}=\beta_{MF}c_{1}\left( 1-ef_{1} \right)y_{1}\frac{y_{6}}{{TOT}_{c}}-(\alpha_{1}+\eta)y_{3}$ Infected not reached FSW (3)

$\frac{dy_{4}}{dt}=\beta_{MF}c_{1}\left( 1-ef_{2} \right)y_{2}\frac{y_{6}}{{TOT}_{c}}-(\alpha_{1}+\eta)y_{4}$ Infected reached FSW (4)

$\frac{dy_{5}}{dt}={\alpha_{2}}{TOT}_{C}+\eta y_{6}-\beta_{FM}c_{2}\left( 1-ef_{1} \right)y_{5}[\frac{y_{3}}{{TOT}_{F1}+{TOT}_{F2}}]-{\beta_{FM}c}_{2}\left( 1-ef_{2} \right)y_{5}\left[ \frac{y_{4}}{{TOT}_{F1}+{TOT}_{F1}} \right]-{{{\alpha_{2}}}y}_{5}$ Susceptible clients (5)

$\frac{dy_{6}}{dt}=\beta_{FM}c_{2}\left( 1-ef_{1} \right)y_{5}\left[ \frac{y_{3}}{{TOT}_{F1}+{TOT}_{F2}} \right]+\beta_{FM}c_{2}\left( 1-ef_{2} \right)y_{5}\left[ \frac{y_{4}}{{TOT}_{F1}+{TOT}_{F2}} \right] -(\alpha_{2}+\eta)y_{6}$ Infected clients (6)

**Model parameterisation**

The model was parameterised and calibrated to a typical *Avahan* district, Bellary, whose HIV prevalence amongst FSWs (15.6%) and clients (6.2%) is around the midpoint of the range of prevalence found in other districts [6-7]. Uncertainty ranges were defined for the behavioural parameters using data from the Integrated bio-behavioural assessment (IBBA) surveys undertaken amongst FSWs and their clients in Bellary [6-7], including IBBA data from other districts for parameterising condom use, whereas the biological parameters used data from the published literature. The total number of FSWs reached per year in this typical district was set to be the mean number of FSWs reached per year (1429 FSWs) over 2004-2007 across all *Avahan* districts. Because the mapping size estimates for the number of FSWs in each district were frequently less than this coverage estimate, the total number of FSWs in the typical district was set to be the mean maximum number of FSWs reached per year (3200) in each district from 2004 to 2011. This suggested *Avahan* reached on average 45% of FSWs in the typical district over the intervention period. The modeled HIV epidemic was assumed to start in 1987 in line with published data suggesting the Indian HIV epidemic started in the late 1980s [8]. The HIV epidemic was started by seeding the FSW population with 1% HIV prevalence, and the system of equations were solved in MATLAB using the ode45solver with a variable time step Runge-Kutta method [9-10].

The calibration of the model was done using the Powell’s method [11] (or Powell’s conjugate direction method) for finding a minimum of a function. Using Scientific Python we employed this gradient descent method to minimize the difference between the model simulated and observed HIV prevalence among FSWs (15.6%) and clients (6.2%) from the Bellary round 1 IBBA in 2004[7]. The prevalence values for FSW ($p_{FSW}$) and clients ($p_{c}$) from the model were calculated as the proportion of the infected FSWs/clients out of the total population of FSWs and clients, i.e.:

$$p_{FSW}=100*\frac{y_{3}+y_{4}}{{TOT}_{F1}+{TOT}_{F2}}, p_{c}=100*\frac{y_{5}}{{TOT}_{c}}$$

To fit the model to observed prevalence in Bellary (full line and dot on Figure S1(a)-(b)) the HIV transmission probability, duration of being a FSW or client and frequency of commercial sex were varied. The calibrated model gave us a set of parameters (in Table S1) for which the modelled HIV epidemic resembled the HIV epidemic in Bellary. We used this set of parameters to describe a typical *Avahan* district. Within this setting we explored how the impact and cost of an *Avahan*-style intervention are influenced by changes in intervention scale (number of FSWs reached per year) and intervention intensity (proxied by number of condoms distributed per reached FSW per year – see appendix S2).

**Impact of Avahan intervention**

The modelled Avahan intervention induced an increase in %CCU among FSWs reached by the intervention ${(so f}_{2}>f_{1})$ by an amount that is related to the intensity of the intervention (Figure 1(b) in the main text). Within the model the overall infection rates for clients ( $I_{clients}$) and FSWs ($I_{FSWs}$) are given by:

$I_{clients}=\beta_{FM}c_{2}y_{5}[ \left( 1-ef_{1} \right)\left( \frac{y_{3}}{{TOT}_{F1}+{TOT}_{F2}} \right)+\left( 1-ef_{2} \right)\left( \frac{y_{4}}{{TOT}_{F1}+{TOT}_{F2}} \right) ]$ (8)

$I_{FSWs}= \beta_{MF}c_{1}[\left( 1-ef_{1} \right)y_{1}\frac{y_{6}}{{TOT}_{c}}+\left( 1-ef_{2} \right)y_{2}\frac{y_{6}}{{TOT}_{c}}]$ (9)

with $f_{1}=f_{2}$ before and in the absence of intervention and $f_{1}\neq f_{2}$ in presence of the intervention. The number of new HIV infections acquired from 2004 to 2007 (Inf) was calculated as the integral over time of these rates:

$Inf=\int_{t=2004}^{2007} {(I}_{clients}+I_{FSWs})dt$ (10)

The impact of the modelled FSW targeted *Avahan* intervention was projected as the number of HIV infections averted due to the presence of the intervention over the 4 year period (2004-2007). This represented the difference in the expression from equation (10) when $f_{1}=f_{2}$ and when $f_{1}\neq f_{2}$ with *Avahan*. The impact is projected in terms of scale (number of FSWs reached between 2004 and 2007 - ${TOT}_{F2}$) and the intervention intensity (annual number of condoms distributed per FSWs reached – determines $f_{2}(t)$) (Figure 3(a) in the main text). Different combinations of scale and intensity can give the same impact. We use this relationship between impact, scale and intensity, in conjunction with the explicit relationship between cost, scale and intensity of the *Avahan i*ntervention to explore the interplay between the key components of the intervention: cost, impact, scale and intensity.

**A2: Sensitivity Analysis**

A sensitivity analysis was undertaken to check the robustness of the model’s projections for the optimal impact achieved for different budgets. All sensitivity analyses considered the effect of changes in model parameters or structure on the projected maximum impact achieved for different budgets, and the scale/intensity combination required to attain this. We also considered how these changes effected the reduction in budget that can occur while maintaining the same impact in the typical/representative Avahan district. We firstly explored (section A2i) the effect of uncertainty in the relationship between the level of consistent condom use amongst reached FSWs (%CCU) and intervention intensity #CD within the 95% CI shown in Figure 1(b). Secondly (section A2ii), we explored how uncertainty in the cost function alters the results. Then, we considered the effect on our model projections of successively altering the model, firstly so that it did not replace HIV deaths but included population growth (section A2iii), secondly by also incorporating an initial acute stage of HIV with increased HIV infectivity (section A2iv), and lastly by also assuming the start of the intervention was delayed until the start of 2014 while incorporating current levels of ART with reduced infectivity and increased survival and not replacing HIV deaths (section A2v). The ART scenario assumed ART started in 2006.

**A2i: Robustness of the optimal intervention projections to uncertainty in the relationship between condom use among reached FSWs and intervention intensity**

We varied the intervention exposure curve from Figure 1(b) in the main text, by considering the upper and lower 95% CI curves shown in Figure 1(b). The rest of the analysis was the same as for the simple baseline model described in the main text for projecting the maximum impact achieved for different budgets, and the scale/intensity combination required to attain this. Our results suggest that the optimal intervention is robust to these changes (Figure S2(b)) and hence the maximum impact attained at any budget is similar (Figure S2(a)). For the typical/representative district, a reduction in budget of 1.9% or 3.1% was possible whilst attaining the *Avahan* impact.

**A2ii: Robustness of the optimal intervention projections to uncertainty in the relationship between cost, scale and intensity**

We varied the coefficients *a* and *b*, that describe the effect of scale and intensity on the cost function in Appendix C within their 95% CI, and repeated the baseline analysis. Our results suggest that with changes to the coefficients, the optimal intervention for different budgets remains qualitatively the same: increase intensity, followed by scaling up and then increase intensity again (Figure S3(b)). As a consequence the relationship between maximum impact and budget remains qualitatively the same (Figure S3(a)) for different cost functions but with variations in the cost for different levels of impact. For the Avahan typical/representative district (scale=1429, intensity=267), a reduction in budget between 1.9% and 3.6% was possible for different $a$ and $b$ whilst maintaining the same Avahan impact.

**A2iii: Robustness of the optimal intervention projections to not replacing HIV deaths in the model**

We adjusted our model equations (1)-(6) to incorporate variable population size, rather than constant population size as assumed in the baseline model analysis. To achieve this, we omitted the terms $\eta y_{3}$ in equation (1), $\eta y_{4}$ in equation (2) and $\eta y_{6}$ in equation (5) and also multiplied the recruitment terms in equations (1), (2) and (5) by a growth factor $\mu$ . This was about 1.03 per year corresponding to the reported increase in population in Bangalore over the period 2001 to 2011 [12]. With this change the model still fit the HIV prevalence data from Bellary and so the model was not refit. Our projections for the necessary combination of scale and intensity to maximise impact at any budget remained the same (comparing the full and the dotted lines in Figures S4(a)-(b))). A reduction of 2.8% in the Avahan budget was projected for the typical/representative district if the intervention’s scale and intensity were optimised whilst still maintaining the impact achieved in the Avahan typical/representative district.

**A2iv: Robustness of the optimal intervention projections to incorporating an acute and chronic stage HIV infection into the model**

**New model equations for A2iv**

$\frac{dy_{1}}{dt}={\alpha_{1}}\mu{TOT}_{F1}-\beta_{MF}c_{1}\left( 1-ef_{1} \right)y_{1}\frac{[y_{6}+{\theta z}_{3}]}{{TOT}_{c}}-\alpha_{1}y_{1}$ susceptible FSWs uncovered (12)

$\frac{dz_{1}}{dt}=\beta_{MF}c_{1}\left( 1-ef_{1} \right)y_{1}\frac{{[y}_{6}+\theta z_{3}]}{{TOT}_{c}}-\Omega z_{1}-\alpha_{1}z_{1}$ infected FSWs **acute** stage uncovered (13)

$\frac{dy_{3}}{dt}=\Omega z_{1}-(\alpha_{1}+\eta)y_{3}$ infected FSWs **chronic** uncovered (14)

$\frac{dy_{2}}{dt}={{\alpha_{1}}\mu TOT}_{F2}-\beta_{MF}c_{1}\left( 1-ef_{2} \right)y_{2}\frac{{[y}_{6}+\theta z_{3}]}{{TOT}_{c}}-{{\alpha_{1}}y}_{2}$ susceptible FSWs covered (15)

$\frac{dz_{2}}{dt}=\beta_{MF}c_{1}\left( 1-ef_{2} \right)y_{2}\frac{[y_{6}+\theta z_{3}]}{{TOT}_{c}}-\Omega z_{2}-\alpha_{1}z_{2}$ infected FSWs **acute** stage covered (16)

$\frac{dy_{4}}{dt}=\Omega z_{2}-{(\alpha}_{1}+\eta)y_{4}$ infected FSWs **chronic** stage covered (17)

$\frac{dy_{5}}{dt}={\alpha_{2}}\mu{TOT}_{C}-\beta_{FM}c_{2}\left( 1-ef_{1} \right)y_{5}\left[ \frac{{\theta z}_{1}+y_{3}}{{TOT}_{F1}+{TOT}_{F2}} \right]-\beta_{FM}c_{2}\left( 1-ef_{2} \right)y_{5}[\frac{\theta z_{2}+y_{4}}{{TOT}_{F1}+{TOT}_{F2}}]-{\alpha_{2}y}_{5}$

susceptible clients (18)

$\frac{dz_{3}}{dt}$ $=\beta_{FM}c_{2}\left( 1-ef_{1} \right)y_{5}\left[ \frac{{\theta z}_{1}+y_{3}}{{TOT}_{F1}+{TOT}_{F2}} \right]+\beta_{FM}c_{2}\left( 1-ef_{2} \right)y_{5}\left[ \frac{\theta z_{2}+y_{4}}{{TOT}_{F1}+{TOT}_{F2}} \right]-\Omega z_{3}-\alpha_{2}z_{3}$ infected clients **acute** stage (19)

$\frac{dy_{6}}{dt}=\Omega z_{3}-(\alpha_{2}+\eta)y_{6}$ infected clients **chonic** stage (20)

Initial conditions:

$y_{1}(0)=0.99*TOT_{F1}, y_{2}\left( 0 \right)=0.99*TOT_{F2},$ susceptible FSWs (unreached and reached by the intervention)

$y_{3}\left( 0 \right)=z_{1}\left( 0 \right)=0.005*TOT_{F1},y_{4}\left( 0 \right)=z_{2}\left( 0 \right)=0.005*TOT_{F2}$infected FSWs (unreached and reached by the intervention)

$y_{5}\left( 0 \right)=TOT_{C},y_{6}\left( 0 \right)=z_{3}\left( 0 \right)=0.$ susceptible and infected clients

New parameters for A2iv adapted model:

$$\Omega=6 per year , \theta=25 times$$

Where $\Omega$ is the leaving rate from the acute stage to the chronic stage and $\theta$ is how much more infectious the acute stage is compared to the chronic stage [13].

Assumptions for acute stage model:

- The acute stage of infection is modelled by splitting the infected populations (both FSWs and clients) into individuals with either acute or chronic HIV infection
- FSWs/clients in the acute stage of infection are more infectious (by 25 time more [13])
- A proportion of infected FSWs in the acute stage are recruited to the chronic infected FSW stage at a rate $\Omega=6 per year$. This is based on assuming that the HIV acute stage lasts about 2 months [13].
- As with A2iii, HIV deaths are not replaced but there is natural growth of the population at 3% per year.

**Model analysis**

We repeated the analysis done for the simple baseline model, although the model (equations (12)-(20)) was firstly re-calibrated to the prevalence in Bellary in 2004 (dash-dot lines in Figure S1(a)-(b)). In order to fit the model, we needed to change the transmission probabilities ($\beta_{MF}$ from 0.006 in the baseline/simple model to 0.0021 here; $\beta_{FM}$ from 0.005905 to 0.00178) and the HIV death rate ($1/\eta$ from 10.3 years to 11.0 years). The rest of the model parameters stayed the same and the additional parameters ($\Omega$ and $\theta$) were fixed to the values given above.

This model projected the same relationship between scale and intensity for the optimal interventions that maximise impact at different budgets (Figure S4(b)). However, the maximum impact achieved for each budget was reduced (Figure S4(a)) because the epidemic was already decreasing without the intervention, and so less infections could be averted than in the baseline simulations. A reduction of 2.8% in the *Avahan* budget was projected for the typical/ representative district if the intervention’s scale and intensity were optimised whilst still maintaining the impact of *Avahan*.

**A2v: Robustness of the optimal intervention projections to delaying the intervention till 2014 whilst incorporating current levels of ART**$.$

**New model equations for A2v**

$\frac{dy_{1}}{dt}={\alpha_{1}}\mu{TOT}_{F1}-\beta_{MF}c_{1}\left( 1-ef_{1} \right)y_{1}\frac{[y_{6}+{\theta z}_{3}+\delta w_{3}]}{{TOT}_{c}}-\alpha_{1}y_{1}$susceptible uncovered FSWs (21)

$\frac{dz_{1}}{dt}=\beta_{MF}c_{1}\left( 1-ef_{1} \right)y_{1}\frac{{[y}_{6}+\theta z_{3}+\delta w_{3}]}{{TOT}_{c}}-\Omega z_{1}-\alpha_{1}z_{1}$ acute infected uncovered FSWs (22)

$\frac{dy_{3}}{dt}=\Omega z_{1}-(\alpha_{1}+{\eta+}\pi)y_{3}$ chronic infected uncovered FSWs (23)

$\frac{dw_{1}}{dt}=\pi y_{3}-{(\alpha}_{1}+\rho\eta)w_{1}$ on ART infected uncovered FSWs (24)

$\frac{dy_{2}}{dt}={{\alpha_{1}}\mu TOT}_{F2}-\beta_{MF}c_{1}\left( 1-ef_{2} \right)y_{2}\frac{{[y}_{6}+\theta z_{3}+\delta w_{3}]}{{TOT}_{c}}-{{\alpha_{1}}y}_{2}$ susceptible covered FSWs (25)

$\frac{dz_{2}}{dt}=\beta_{MF}c_{1}\left( 1-ef_{2} \right)y_{2}\frac{[y_{6}+\theta z_{3}+\delta w_{3}]}{{TOT}_{c}}-\Omega z_{2}-\alpha_{1}z_{2}$ acute infected covered FSWs (26)

$\frac{dy_{4}}{dt}=\Omega z_{2}-{(\alpha}_{1}+\eta+\pi)y_{4}$ chronic infected covered FSWs (27)

$\frac{dw_{2}}{dt}=\pi y_{4}-{(\alpha}_{1}+\rho\eta)w_{2}$ on ART infected covered FSWs (28)

$\frac{dy_{5}}{dt}={\alpha_{2}}{\mu TOT}_{C}-\beta_{FM}c_{2}\left( 1-ef_{1} \right)y_{5}\left[ \frac{{\theta z}_{1}+y_{3}+\delta w_{1}}{{TOT}_{F1}+{TOT}_{F2}} \right]-\beta_{FM}c_{2}\left( 1-ef_{2} \right)y_{5}[\frac{\theta z_{2}+y_{4}+\delta w_{2}}{{TOT}_{F1}+{TOT}_{F2}}]-{\alpha_{2}y}_{5}$

susceptible clients (29)

$\frac{dz_{3}}{dt}$ $=\beta_{FM}c_{2}\left( 1-ef_{1} \right)y_{5}\left[ \frac{{\theta z}_{1}+y_{3}+\delta w_{1}}{{TOT}_{F1}+{TOT}_{F2}} \right]+\beta_{FM}c_{2}\left( 1-ef_{2} \right)y_{5}\left[ \frac{\theta z_{2}+y_{4}+\delta w_{2}}{{TOT}_{F1}+{TOT}_{F2}} \right]-\Omega z_{3}-\alpha_{2}z_{3}$ acute infected clients (30)

$\frac{dy_{6}}{dt}=\Omega z_{3}-(\alpha_{2}+\eta+\pi)y_{6}$ chronic infected clients (31)

$\frac{dw_{3}}{dt}=\pi y_{6}-\left( \alpha_{2}+\rho\eta\right)w_{3}$ on ART infected clients (32)

Initial conditions:

$$y_{1\left( 0 \right)}=0.99*TOT_{F1}, y_{2}\left( 0 \right)=0.99*TOT_{F2},y_{3}\left( 0 \right)=z_{1}\left( 0 \right)=0.005*TOT_{F1},y_{4}\left( 0 \right)=z_{2}\left( 0 \right)=0.005*TOT_{F2}, y_{5}\left( 0 \right)=TOT_{C},y_{6}\left( 0 \right)=z_{3}=0,w_{1}=w_{2}=w_{3}=0.0$$

New parameters for A2v adapted model:

$$\Omega=6 per year , \theta=25 times, \delta=0.1,\rho=0.33$$

Here $\Omega$ is the recruitment rate from the acute to chronic stage of HIV, $\pi$ is the recruitment rate from chronic HIV stage to ART, $\theta$ is the factor increase in infectivity during the acute stage compared to chronic stage of HIV, $\delta$ is the reduction in infectivity whilst on ART and $\rho$ is the reduction in HIV mortality rate when on ART.

**Assumptions for the ART model:**

-The model is the same as the model including acute infection except there is now an ART group for covered and uncovered FSWs and clients.

-Individuals are recruited on to ART from the chronic infected groups at rate $\pi$ which is set to zero before 2006 when no ART was provided in South India. After 2006, it is given a non-zero constant value that is fit to give 20% of HIV infected individuals being on ART by end of 2012 [14]. This $\pi$ value that gave this coverage of ART in 2012 was 5.7% per year.

-FSWs on ART are less infectious [14] ($\delta$=10% of the infectivity of those with chronic infection) and have reduced mortality ($\rho=0.33$).

We used the same parameters as for the calibrated model from appendix A2iv to parameterise the model for A2v, but then kept condom use constant from 2003 until the start of 2014 ($f_{2}$ and $f_{1}$ stay constant at their 2003 values) on the assumption that the intervention has been delayed until then. The model was then used to consider the same intervention starting in 2014 and evaluated over the next 4 years, but now with ART at high coverage. The model was again used to project the maximum impact achieved for different budgets, and the scale/intensity combination required to attain this. We also considered how these changes effected the reduction in budget that can occur while maintaining the same impact in the typical *Avahan* district.

When the intervention is delayed whilst incorporating current coverage levels of ART, our projections for the optimal intervention combination at different budget levels remain the same. However, as with the model including the acute stage, although the relationship between the optimised impact at different budget level does not change qualitatively the impact achieved at any specific budget level is reduced (dashed lines in Figure S4(a)-(b)). This is due to similar reasons as for the model incorporating acute HIV infection – i.e. the model suggests the HIV epidemic is in decline even without *Avahan*. Lastly, the model suggests the budget in the typical/representative district could reduce by 3.1% whilst maintaining the same impact if scale and intensity had been optimised.
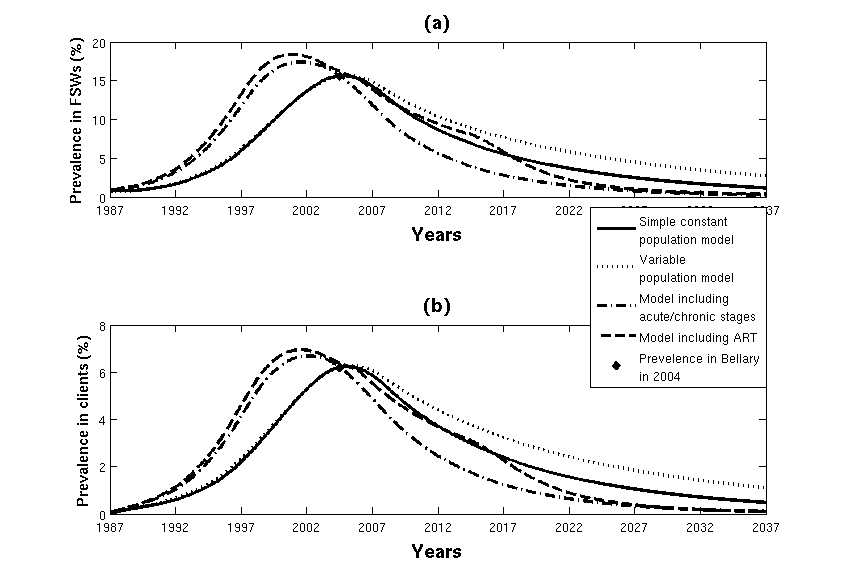


**Figure S1**: The HIV prevalence over time amongst FSWs (a) and their clients (b) for different models. For each, the scale was fixed to the average across all Avahan districts over 2004-2007 (1429 reached FSWs), and the intensity corresponded to #CD=267 condoms distributed per reached FSW per year. The rest of the model parameters are as in Table S1.


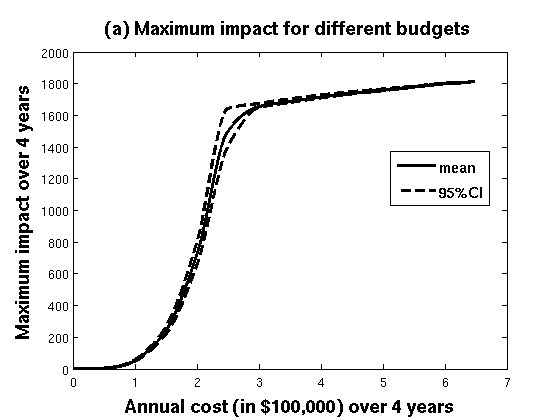

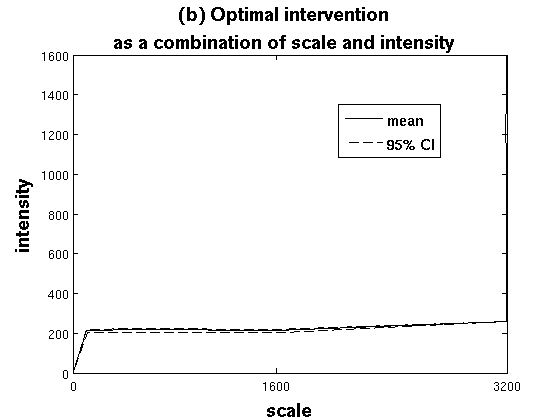


**Figure S2**: Robustness of the relationship between incremental annual cost and impact over 4 years (a) or scale and intensity (b) for the optimal intervention strategies that maximise impact for different budget levels. We show this for uncertainty in condom exposure function from Appendix B. Model parameters remain as per Table S1 and we vary the relationship between #CD and $f_{2}(2008)$ as per Figure 1(b) in the main text.


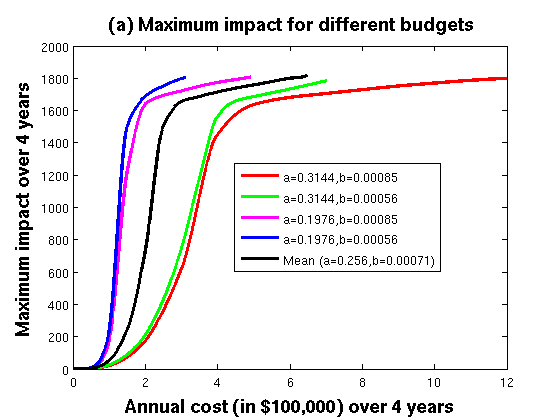

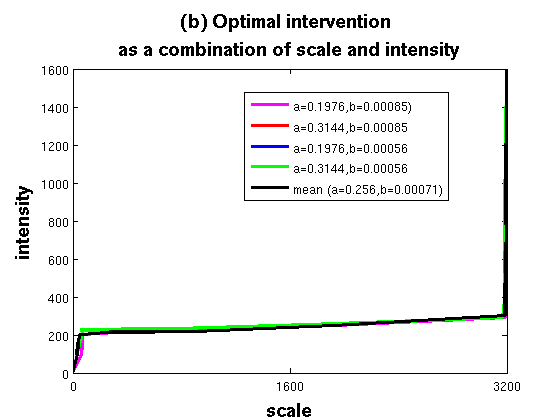


**Figure S3**: Robustness of the relationship between incremental annual cost and impact over 4 years (a) or scale and intensity (b) for the optimal intervention strategies that maximise impact for different budget levels. We show this for uncertainty in cost function coefficients in equation A1. Model parameters remain as per Table S1 and we vary the coefficients a and b in the cost functions $TC={scale}^{a}e^{10.18+b*intensity}$ within their 95%CI: a=(0.1976,0.3144); b=(0.00056,0.00085) with the mean a=0.256 and mean b=0.00071.


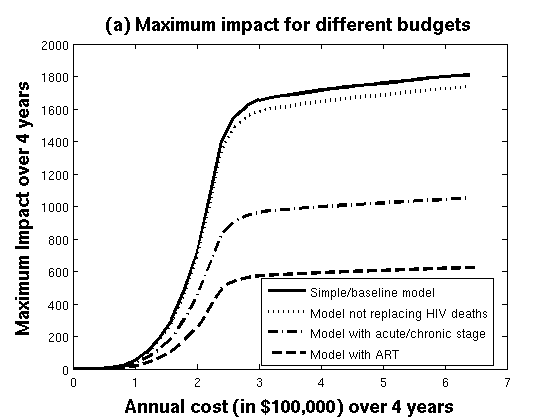

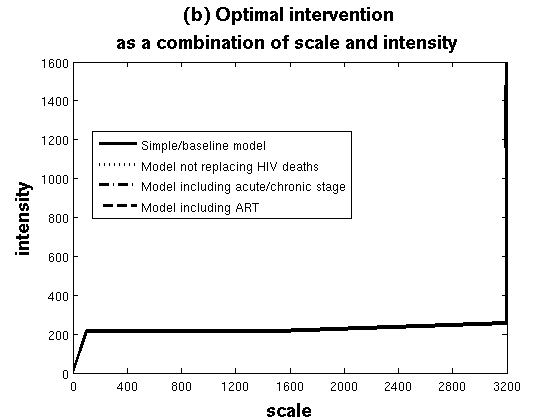


**Figure S4**: Robustness of the relationship between incremental annual cost and impact over 4 years (a) or scale and intensity (b) for the optimal intervention strategies that maximise impact for different budget levels. We project this relationshp when we consider 4 different model changes. The parameters remain as per Table S1 with additional parameters descibed in section A2iv.

**Table S1**: Model parameter definitions, symbols, ranges and references

| **Parameter description** | **Symbol** | **Model value** | **Reference** |
| --- | --- | --- | --- |
| Average duration of HIV from initial infection to death (the same for both FSWs and clients) in years | $1/\eta$ | 10.1 (8.6-10.8) | ^[18,19]^ |
| Probability of HIV transmission from female (FSWs) to male (clients) per sex act | $\beta_{FM}$ | 0.00600 (0.0006-0.006) | ^[4,15]^ |
| Probability of HIV transmission from male (clients) to female (FSWs) per sex act | $\beta_{MF}$ | 0.005905 (0.0006-0.006) | ^[4,15]^ |
| Number of clients per FSW per year using their reported clients had sex with on last day * number of days entertain clients in a typical month (per year) | $c_{1}$ | 438.48 (95% CI 368.76 – 568.32) | ^[6,7]^ |
| Number of FSWs per client per year using client reported FSWs in the last month | $c_{2}$ | 24.8 (95% CI 20.52-23.4) | ^[6,7]^ |
| Efficacy of condoms (vaginal sex) | e | 0.85 (0.66-0.94) | ^[16]^ |
| Duration as a client in years | ${1/\alpha}_{2}$ | 7.063 (7.58-9.92) | ^[6,7]^ |
| Current duration as a FSW in years | $1/\alpha_{1}$ | 5.762 (4.1-15.75) | ^[6,7]^ |
| Total population of FSWs | ${TOT}_{F}$ | 3200 | ^[6,17]^ |
| Coverage level of Avahan | m | m=0 before Avahan and m=[0,1] for the duration of Avahan | model variable |
| Total population of FSWs not covered by the intervention | ${TOT}_{F1}$ | A proportion (1-m) of ${TOT}_{F}$ | model variable |
| Total population of FSWs covered by the intervention | ${TOT}_{F2}$ | A proportion m of ${TOT}_{F}$;m=[0,1] | model variable |
| Total client population | ${TOT}_{C}$ | ${TOT}_{c}=\frac{{c_{1}TOT}_{F1}+c_{2}{TOT}_{F2}}{c_{3}}$ | determined by other model parameters |
| Prevalence of FSWs in rd1 (Bellary) | $p_{FSW}$ | 15.6% (11.1-20.0) | ^[6]^ |
| Prevalence in clients in rd 1 (Bellary) | $p_{C}$ | 6.2%(2.6-9.5) | ^[6]^ |
| Year of seeding the HIV infection |  | 1987 | ^[8]^ |
| HIV seeded prevalence in FSWs |  | 1% | model assumption |
| HIV seeded prevalence in clients |  | 0% | model assumption |

Typical district: scale=1429 (45% of 3200 reached), intensity=267

**References for Appendix S1:**

1. Bill and Melinda Gates Foundation (2008) Avahan - The India AIDS Initiative: The business of HIV prevention at scale. Accessed 2014 August 24. <http://docs.gatesfoundation.org/avahan/documents/avahan_hivprevention.pdf>
2. Bill & Melinda Gates Foundation (2010), Avahan India AIDS Initiative: Common Minimum Program. Accessed 2014 August 24. <http://docs.gatesfoundation.org/avahan/documents/cmp-monograph.pdf>
3. Vickerman P, Foss AM, Pickles M, Deering K, Verma S, et al.(2010) To what extent is the HIV epidemic in southern India driven by commercial sex? A modelling analysis. AIDS 24: 2563–2572.
4. Boily MC, Pickles M, Lowndes CM, Ramesh BM, Washington R. et al. (2013) Positive impact of large-scale HIV prevention programme among female sex workers and clients in South India. AIDS 27(9):1449-1460.
5. Pickles M, Foss A, Vickerman P, Deering K, Verma S, et al. (2010) Interim modelling analysis to validate reported increases in condom use and assess HIV infections averted among female sex workers and clients in southern India following a targeted HIV prevention program. Sex Transm Infect 2010; 86 (1):33–43.
6. Ramesh BM, Moses S, Washington R. (2008) Determinants of HIV prevalence among female sex workers in four south Indian states: analysis of cross-sectional surveys in twenty-three districts. AIDS. 22(Suppl 5):S35–44.
7. National Summary Report (December 2009), India Integrated Behavioural and Biological Assessment (IBBA), Round 1 (2005-2007) , Indian Council of Medical Research & Family Health International. Accessed 2014 August 24. http://www.ibbainfo.in/keydoc/reports/NSRR1.pdf
8. Arora P, Cyriac A, Jha P. (2004) India’s HIV-1 epidemic. CMAJ 171:1337–1338.
9. Bogacki, P,Shampine LF. (1989) A 3(2) pair of Runge-Kutta formulas. Appl. Math. Letters 2:321–325.
10. Dormand J R,Prince PJ. (1980) A family of embedded Runge-Kutta formulae. J. Comp. Appl. Math. 6:19–26.
11. Powell MJD. (1964). An efficient method for finding the minimum of a function of several variables without calculating derivatives. Computer Journal **7**(2): 155–162.
12. Bangalore District Census 2011. Accessed 2014 August 24

http://www.census2011.co.in/census/district/242-bangalore.html

1. Hollingsworth TD, Anderson RM, et al. (2008) HIV-1 transmission, by stage of infection. J. Infect. Dis. 198(5): 687-693
2. Mishra S, Mountain E, Pickles M, Vickerman P, Shastri S, et al. (2014) Exploring the population-level impact of antiretroviral treatment: the influence of baseline intervention context. AIDS.28(1):S61-72.
3. Boily MC, Baggaley RF, Wang L, Masse B, White RG et al. (2009) Heterosexual risk of HIV-1 infection per sexual act: a systematic review and meta-analysis of observational studies. Lancet Infect Dis. 9(2): 118-129.
4. Pinkerton SD, Abramson PR, Turk ME. (1998) Updated estimates of condom effectiveness. J Assoc Nurses AIDS Care 9:88–89.
5. National Summary Report (Mach 2011), India Integrated Behavioural and Biological Assessment (IBBA), Round 2 (2009-2019), Indian Council of Medical Research & Family Health International. Accessed 2014 August 24. http://www.ibbainfo.in/reports.php
6. Grover G, Shivraj SO (2004) Survival pattern of reported HIV infected individuals in the city of Delhi (India). J Commun Dis **36**:83-92.
7. Kumarasamy N, Solomon S, Flanigan TP, Hemalatha R, Thyagarajan SP (2003) Natural history of human immunodeficiency virus disease in southern India. Clin Infect Dis **36**:79-85.
