# Supplementary material for: Optimal Allocation of Resources in Female Sex Worker Targeted HIV Prevention Interventions: Model Insights from Avahan in South India
Source: PLoS One. 2014 Oct 1;9(10):e107066. doi: 10.1371/journal.pone.0107066 (PMC4182672; doi:10.1371/journal.pone.0107066)
Supplement: Appendix S2 — Description and analysis of the Integrated Biological and Behavioural Assessment (IBBA) survey data to estimate the level condom use among reached FSWs by Avahan for different levels of intervention intensity. (DOCX) [file pone.0107066.s002.docx]

**Appendix S2: Description and analysis of the Integrated Biological and Behavioural Assessment (IBBA) survey data to estimate the level condom use among reached FSWs by *Avahan* for different levels of intervention intensity**

**Overview**

We used data available from *Avahan* [1-2], the India AIDS initiative, to establish a relationship between the level of condom-use among *Avahan*-reached FSWs and the intensity of service delivery of the *Avahan* intervention. This data is freely available from http://ibbainfo.in/. We also used this data to reconstruct a linear time-trend for the increase of condom use among all FSWs before, during and after *Avahan* (1987-2008, see Figure 1(a) in the main text). These relationships (Figures 1(a,b) from the main text) were used as an input to the mathematical model described in Appendix S1, specifically to project the impact of the intervention as a function of the intervention’s intensity of service delivery during 2004-2007.

**Introduction**

In 2003, the Bill & Melinda Gates Foundation established Avahan [1-2], the India AIDS initiative, to target the high-risk groups that were driving the HIV epidemic in India. These included female sex workers (FSWs) and their commercial partners (clients), men who have sex with men (MSM) and people who inject drugs (IDUs). The aim of the *Avahan* initiative was to reduce the HIV prevalence in these groups, and therefore prevent further transmission to the general population [3].

Rollout of *Avahan* programme activities began in January 2004 [1,4] reaching almost all districts by mid-2005 with rapid scale-up within each district. By December 2008, *Avahan* had established a large-scale, targeted HIV preventive intervention with more than 75% of the estimated target population of female sex workers (total population 217 000) being contacted monthly [4-6].

Through state-level providers (SLPs) and local non-governmental organisations (NGOs), *Avahan* worked in 64 districts in four states of South India (Andhra Pradesh, Karnataka, Maharashtra, and Tamil Nadu). The primary data collected as part of the *Avahan* evaluation were the serial cross-sectional integrated behavioural and biological assessment surveys [4,5,7] (IBBAs) done among FSWs, their clients, and MSM in 24 districts, referred to as IBBA districts. IBBA districts contain about 38% of the FSWs and 45% of the high-risk MSM across the 69 *Avahan* districts in South India. There were up to 3 rounds of IBBA surveys undertaken, with the first IBBA (round 1) being conducted 7-19 months after *Avahan* initiation [4], whereas the round 2 and round 3 follow-up surveys were undertaken 28–37 and 56–73 months after round 1, respectively [5,7].

In this appendix, we describe some statistical analyses that was undertaken to feed into our main modelling analyses. The analyses used data from the IBBA surveys conducted amongst FSWs from four districts (Bangalore, Belgaum, Bellary and Shimoga) within the state of Karnataka in Southern India over rounds 1 and 2 between 2004 and 2008. This data is freely available from <http://ibbainfo.in/>. The purpose of the analyses was to determine the relationship between a FSW’s intensity of exposure to the *Avahan* intervention and their level of self-reported condom use with their clients in 2008, and also to establish a time trend in the level of condom use among FSWs that were reached by *Avahan* or not.

**Summary of analysis**

Our analyses focussed on four districts (Bangalore, Belgaum, Bellary and Shimoga) within the state of Karnataka in Southern India. The target sample size per district was generally 400 completed interviews and blood samples, although in Bangalore the sample was increased to 800, to account for different sex work typologies and because it is a larger urban area. Three different sampling approaches were used including cluster sampling primarily for brothel-based FSWs and those in homes and lodges, and time-location cluster sampling for street-based FSWs. Additional details on the sampling methods can be found in Ramesh et al. 2008 [7].

The IBBA data in our analysis was used to establish a relationship between the level of condom use amongst reached FSWs and the intensity of *Avahan.* The level of condom use among FSWs was defined by the average % of FSWs using condoms in their last sexual act (%CCU) because this translated well to what is used in the model – average consistency or probability of condom use for each sex act. We then used available survey data to establish a relationship between %CCU amongst the *Avahan*-reached FSWs and different measures of the intensity of *Avahan* in 2008.

There were several possible measures of the Avahan intervention’s intensity of service delivery: monthly number of condoms distributed to each FSW from non-governmental organisations (NGO) (#CD); monthly number of staff contacts per FSW; and monthly number of condom demonstrations per FSW organised by the NGOs. However, because #CD was the only intervention intensity measure for which a cost function could be estimated from *Avahan* data (see Appendix S3), #CD was chosen as the proxy measure of intervention intensity considered in the following statistical analyses and used in all model analyses. Logistic regression analysis showed that #CD was positively correlated to %CCU after controlling for potential confounders (see next section for details), with increased %CCU among FSWs that report obtaining more condoms (#CD) (as per Figures 1(b) in the main text). It is important to emphasise that this relationship between %CCU and #CD does not just represent the effect of distributing more condoms to FSWs, but that it is also a proxy for increases in other measures of intervention intensity as evident by the correlation between the logarithm of #CD and the monthly number of condom demonstrations per FSW (correlation coefficient β=0.25, p<0.001) or the monthly number of staff contacts (correlation coefficient β=0.1, p<0.001). Also, because condoms were available from other sources, %CCU should not be expected to be zero when #CD is zero.

**Specific methods for the regression analysis between condom use and #CD**

With intervention intensity proxied by the annual number of condoms distributed to each reached FSW per year (labelled #CD) we used the IBBA surveys [4-5] to establish a relationship between #CD and the level of condom use amongst reached FSWs at the end of *Avahan* (c 2008). To do this, multiple questions from the IBBA round 2 dataset [5] (c 2008) for Bangalore, Belgaum, Bellary and Shimoga were used, and the analysis was done in a number of steps. Firstly, individuals were asked if they were aware of the intervention Karnataka Health Promotion Trust (KHPT) or their NGO partners. Those who had not were discounted from the analysis. Secondly, individuals who were in touch with the intervention were then asked if they had ever received condoms from KHPT or their partners. Those who answered positively were included in the final analysis. These two steps ensured that we only included in the analysis those FSWs that have been ‘reached’ by the *Avahan* intervention.

In the next step of the analysis, a new variable was generated to estimate the total number of condoms received by these reached FSWs in a single year by combining the questions: ‘how often are you given condoms by a peer/worker?’ and ‘how many condoms were you given the last time you were given them by a peer/worker?’. Specifically, for the question on ‘how often condoms are given’ multiple answers existed:

- Every day (we assumed condoms were received 365 times per year)

- Once a week (condoms were received 52 times per year)

- More than once a week (condoms were received 104 times per year)

- Fortnightly (condoms were received 26 times per year)

- Once a month (condoms were received 12 times per year)

These numbers were then multiplied by the ‘number received at last time’ to generate an estimate for the total number of condoms received by reached FSWs in a given year or #CD. Estimates for #CD were grouped categorically as follows:

group 0 (baseline group) : 1-144 condoms received per year

group 1:145-300 condoms received per year

group 2:301-600 condoms received per year

group 3:601+ condoms received per year

Each group was then linked to a %CCU with the relationship between #CD and %CCU being shown in Figure S5. Logistic regression analysis was undertaken in STATA version 12 to test for trend in the relationship between %CCU and #CD, while controlling for potential confounders: age (continuous), duration of sex work (continuous), ever married (binary), sex worker typology (categorical), literacy (binary), number of clients in past week who had sexual encounter with (continuous) and whether sex was their main source of income (binary). Table S2 contains the results of the logistic regression.

To use these results in the model analysis, a curve was fit to the relationship between #CD and %CCU using interpolation curve fitting in MATLAB. This curve (Figure 1(b) in the main text) was used to give $f_{2}$ in 2008 depending on the level of intervention intensity #CD. Lower and higher bounds were also fit to the 95% confidence intervals of the data (shown as dashed lines in Figure 1(b) of the main text and within Table S2), and were used in the sensitivity analysis.

**Methods for establishing the time trends of level of condom use among FSWs**

We used these values for %CCU in 2008 (related to #CD in 2008 as per Figure 1(b) in the main text) together with the reported level of condom use of 30.5% in 2003 from the round 1 IBBA FSWs surveys for 4 Karnataka districts (Bangalore, Belgaum, Bellary and Shimoga) [4] to reconstruct a linear time-trend for the increase of condom use among all FSWs before, during and after *Avahan* (1987-2008, see Figure 1(a) in the main text). The analysis undertaken here followed that by Lowndes at al. [8]. Specifically we assumed that until 1997 the reported level of condom use was low (here assumed 0%) and we used reported levels of condom use from rounds 1 and 2 IBBA FSWs surveys in the same 4 Karnataka districts to give us the level of condom use in 2003 (30.5%) and 2008 (depending on #CD varying between 56.1% and 95.6% for the reached FSWs by *Avahan*). We assumed that the level of condom use in 2008 among FSWs not reached was equivalent to the minimum level of condom use among FSWs reached (i.e. 56.1% from group 1 described in the previous section – compares well to the level of condom use amongst unreached FSWs). We then fitted these time points in 1997 (0%), in 2003 (30.5%) and in 2008 (varying between 56.1% and 95.6% for the FSWs reached by *Avahan* and 56.1% for FSWs not reached by *Avahan*) to obtain the time trends reported in Figure 1(a) from the main text.

**Summary**

We used data from IBBA surveys undertaken as part of *Avahan* [4,5,7] to establish a time trend in the level of condom use among reached and unreached FSWs (Figure 1(a) from the main text) and a relationship between the level of condom use among FSWs reached by *Avahan* (%CCU) and the intensity of the *Avahan* intervention (proxied by annual number of condoms distributed per reached FSWs per year over the period 2004-2007=#CD) (Figure 1(b) of the main text). These were used to parameterise the model condom use functions f_1_(t) (unreached FSWs) and f_2_(t) (reached FSWs) (Figure 1(a) in the main text) and to establish the level of condom use in 2008 among FSWs reached by *Avahan* for different levels of intervention intensity of service delivery (Figure 1(b) of the main text).

**Figure S5**: The relationship between number of condoms distributed (different #CD groups on the x axis per month; we multiply by 12 in our analysis to estimate per year) and the level of condom use among FSWs (%CCU on the right y-axis) or the number of FSWs in each #CD group (on the left y-axis).

| Number of condoms distributed in last month (n=1610) | Odds ratio, condom use at last sex act | P>\|Z\| | 95% Conf. Interval |
| --- | --- | --- | --- |
| 0-12 (41) | 0 |  |  |
| 12-25 (97) | 7.4 | 0.0001 | (3.0, 18.1) |
| 25-50 (279) | 10.7 | 0.0001 | (4.9, 23.4) |
| 50+ (1194) | 16.5 | 0.0001 | (8.0, 33.8) |

**Table S2**: Results from multi-linear regression analysis test for trend (controlling for age, duration of sex work, ever being married, sex worker typology, literacy, number of clients in past week and sex work as main source of income – none of which were significant at p<0.05) for number of condoms distributed to female sex workers in the last month and consistency of condom use in last sex act with commercial partner.

**References for Appendix S2:**

1. Bill and Melinda Gates Foundation (2008) Avahan - The India AIDS Initiative: The business of HIV prevention at scale. Accessed 2014 August 24. <http://docs.gatesfoundation.org/avahan/documents/avahan_hivprevention.pdf>
2. Bill & Melinda Gates Foundation (2010), Avahan India AIDS Initiative: Common Minimum Program. Accessed 2014 August 24. <http://docs.gatesfoundation.org/avahan/documents/cmp-monograph.pdf>
3. Bollinger RC, Tripathy SP, Quinn TC. (1995) The human immunodeficiency virus epidemic in India. Current magnitude and future projections. Medicine (Baltimore) 74: 97–106.
4. National Summary Report (December 2009), India Integrated Behavioural and Biological Assessment (IBBA), Round 1 (2005-2007) , Indian Council of Medical Research & Family Health International. Accessed 2014 August 24. http://www.ibbainfo.in/keydoc/reports/NSRR1.pdf
5. National Summary Report (Mach 2011), India Integrated Behavioural and Biological Assessment (IBBA), Round 2 (2009-2019), Indian Council of Medical Research & Family Health International. Accessed 2014 August 24. http://www.ibbainfo.in/reports.php
6. Pickles M, Boily MC, Vickerman P, Lowndes CM, Moses S et al. (2013) Assessment of the population-level effectiveness of the Avahan HIV-prevention programme in South India: a preplanned, causal-pathway-based modeling analysis. The Lancet Glob Health 1(5):289:299.
7. Ramesh BM, Moses S, Washington R. (2008) Determinants of HIV prevalence among female sex workers in four south Indian states: analysis of cross-sectional surveys in twenty-three districts. AIDS. 22(Suppl 5):S35–44.
8. Lowndes CM, Alary M, Verma S, Demers E, Bradley J, et al. (2010) Assessment of intervention outcome in the absence of baseline data: ‘reconstruction’ of condom use time trends using retrospective analysis of survey data. Sex Transm Infect 86 (1):49–55.
