# Supplementary material for: Optimal Allocation of Resources in Female Sex Worker Targeted HIV Prevention Interventions: Model Insights from Avahan in South India
Source: PLoS One. 2014 Oct 1;9(10):e107066. doi: 10.1371/journal.pone.0107066 (PMC4182672; doi:10.1371/journal.pone.0107066)
Supplement: Appendix S3 — Relationship between intervention costs, and intervention scale and intensity of service delivery. (DOCX) [file pone.0107066.s003.docx]

**Appendix S3: Relationship between intervention costs, and intervention scale and intensity of service delivery**

**Overview**

We used data from 64 districts covered by the *Avahan* India AIDS Initiative to establish an explicit formula for the relationship between the total annual cost of the intervention over the period 2004-2007 and two components of the intervention: scale (proxied by the average annual number of FSWs reached between 2004 and 2007) and intensity (proxied by the average annual number of condoms distributed per reached FSW per year between 2004 and 2007). This cost data is available from the authors upon request subject to approval from the relevant Indian authorities.

**Introduction**

The *Avahan* India AIDS Initiative [1-2] represented a large-scale HIV prevention programme supported by the Bill and Melinda Gates Foundation and initiated in 64 districts in four states of South India (Andhra Pradesh, Karnataka, Maharashtra, and Tamil Nadu). Avahan targeted the high-risk groups that were believed to be driving the HIV epidemic in India in the early 2000s [3]. These included female sex workers (FSWs) and their commercial partners (clients), men who have sex with men (MSM) and people who inject drugs (IDUs). The aim of the *Avahan* intervention was, via condom use and promotion activities, to reduce the HIV prevalence in these groups, and prevent further transmission to the general population [4].

*Avahan* was implemented by local non-governmental organisations (NGOs) supported through lead implementing partners at the state level (SLPs) to deliver prevention activities for high-risk and bridge populations in India [5]. The purpose of our analysis was to consider all the costs involved in *Avahan*, and use statistical analysis to determine components of the intervention that mostly affected changes in cost. Furthermore, we expressed total intervention cost per year over the *Avahan* period (2004-2007) in terms of these intervention components and used these relationships in our resource allocation analyses. Specifically, the intervention components we considered were the intervention’s strength or intensity of service delivery and the scale (or coverage) of the intervention.

**Methods**

For our cost study we examined cost data from 64 districts in southern India over the first four years of *Avahan* (2004-2007) and obtained costs from NGOs, SLPs, the Bill and Melinda Gates foundation *Avahan* office, and Pan-*Avahan* capacity building partners – the costs and collection methods are described in detail in Chandrashekar et al. [5-6]. The same costing method was used across all NGOs and over time, limiting bias resulting from heterogeneity in the costing methods. Every NGO involved in the programme was automatically included in the sample, allowing us to have an exhaustive sample of the NGOs in the *Avahan* programme over the period considered. Costs included recurrent costs (personnel costs, project building and operating expenses, travel expenses, STI supplies, monitoring cost, information education & communication, training, condom supplies and indirect expenses) and capital costs (rent, equipment, furniture, vehicle, initial training, insurance and deposits, and start up cost). SLP costs were allocated to NGOs based on interviews and expenditure records. Where they could not be allocated broadly, capital and programme administration costs were shared equally to all their NGOs, and recurrent costs relating to service provision were allocated based on the estimated catchment population size. Bill and Melinda Gates foundation costs were allocated to the SLPs based on their grant size and further down to the NGO based on the estimated population. The Pan-*Avahan* capacity building partner costs were shared equally to all SLPs and further to the NGOs. Capital costs were annualised using a discount rate of 3% and are assumed to have a life of between 5 and 10 years. Economic costs were computed by valuing donated goods at their market price. Prices were adjusted for inflation using the GDP. All costs are presented in US$ 2008.

The scale of the intervention was proxied by the number of FSWs reached per year over the 4 years 2004-2007 (${TOT}_{F2}$ in the model). Different variables could be used to describe the intervention intensity: the number of condoms distributed annually per reached FSW (#CD), the annual number of NGO contacts with the reached FSWs, the annual number of condom demonstrations organised by NGOs etc. However, #CD was the only proxy for the intervention’s intensity of service delivery for which we had sufficient data for each district and year to estimate the total cost. Hence we used this to describe the intensity of the *Avahan* intervention in different districts.

To estimate the coefficients that describe how the total cost of the intervention at the district level (TC) changes with variations in scale and intensity, we used a panel estimator with NGO fixed effects. This accounted for the unobserved characteristics of the NGOs that were time invariant. Regression analyses were conducted using STATA version 13 to derive an empirical equation for the total annual intervention cost over 4 years (TC) in terms of intervention scale (${TOT}_{F2}$) and intervention intensity (#CD).

**Results**

63 *Avahan* districts had data describing the scale and intensity of *Avahan* for 2004-2007. Both were highly variable across districts with on average 1729 (range 0-3200) FSWs reached by *Avahan* each year, and 267 (range 0-1600) condoms being distributed to each reached FSW each year.

For scale varying between 0-3200 and intensity between 0-1600, the statistical regression analysis gave us the coefficients $a$ and $b$ that describe the effect of the logarithm of scale and intensity on the logarithm of the annual total intervention cost in each district. On average over the 4 years they were calculated as a=0.256 (95% CI 0.198-0.314) and b=0.00071 (95% CI 0.00056-0.00085). These coefficients allowed us, with mathematical manipulation to derive the following empirical function for the annual total intervention cost over 4 years (TC) in terms of intervention scale (${TOT}_{F2}$) and intervention intensity (#CD).

$TC\left( {TOT}_{F2},\#CD \right)=({({TOT}_{F2})}^{a}e^{10.18+b(\#CD)}$ (1)

This functional form demonstrates a different relationship exists between the increased costs associated with increasing intervention intensity and the relationship associated with increasing scale (Figure 2(c)-(d) from the main text). Specifically, with increasing scale of the intervention there were clear economies of scale with it costing incrementally less to reach progressively more FSWs (Figure 2(c) from the main text). We found no evidence for diseconomies of scale at high scale. Conversely, the incremental cost of increasing intervention intensity was also non-linear, and showed positive correlation with total cost. We also found that different combinations of scale and intensity could have the same cost (Figure 3(b) in the main text).

**Summary and how we used the results from this work**

Using data from the *Avahan* intervention between 2004 and 2007 we derived an explicit equation for the annual total cost of the *Avahan* intervention over this period in terms of scale (annual number of FSWs reached over the 4 years) and intensity (annual number of condoms distributed per reached FSWs per year). This cost function (equation (1)) was used together with the impact projections from the model to study the relationship between intervention impact, cost, scale and intensity. Specifically, since different combinations of scale and intensity could have the same cost (Figure 3(b) in the main text), we could explore what combination of scale and intensity could maximise impact for different budget levels. Similarly, we could determine the combination of scale and intensity that would minimise cost for a specified impact. This interplay between cost and impact of the intervention, for different combinations of scale and intensity achieved allowed us to make projections of the allocative efficiency of *Avahan*.

**References for Appendix S3:**

1. Bill and Melinda Gates Foundation (2008) Avahan - The India AIDS Initiative: The business of HIV prevention at scale. Accessed 2014 August 24. <http://docs.gatesfoundation.org/avahan/documents/avahan_hivprevention.pdf>
2. Bill & Melinda Gates Foundation (2010), Avahan India AIDS Initiative: Common Minimum Program. Accessed 2014 August 24. <http://docs.gatesfoundation.org/avahan/documents/cmp-monograph.pdf>
3. Bollinger RC, Tripathy SP, Quinn TC. (1995) The human immunodeficiency virus epidemic in India. Current magnitude and future projections. Medicine (Baltimore) 74: 97–106.
4. Pickles M, Boily MC, Vickerman P, Lowndes CM, Moses S et al. (2013) Assessment of the population-level effectiveness of the Avahan HIV-prevention programme in South India: a preplanned, causal-pathway-based modeling analysis. The Lancet Glob Health 1(5):289:299.
5. Chandrashekar S, Vassall A, Reddy B, Shetty G, Vickerman P et al. (2011) The costs of HIV prevention for different target populations in Mumbai, Thane and Bangalore BMC PH 11(6):S7 .
6. Chandrasekaran S, Dallabetta G, Loo V, Mills S, Saidel T (2008) Evaluation design for large-scale HIV prevention programmes: the case of Avahan, the India AIDS initiative. AIDS 22 (5):S1–S15.
